# Harnessing light-activated gallium porphyrins to combat intracellular *Staphylococcus aureus* using an in vitro keratinocyte infection model

**DOI:** 10.1038/s41598-024-84312-4

**Published:** 2025-01-08

**Authors:** Klaudia Szymczak, Michał Rychłowski, Lei Zhang, Joanna Nakonieczna

**Affiliations:** 1 Laboratory of Photobiology and Molecular Diagnostics, Intercollegiate Faculty of Biotechnology, University of Gdansk and Medical University of Gdansk, Gdańsk, Poland; 2 Laboratory of Virus Molecular Biology, Intercollegiate Faculty of Biotechnology, University of Gdansk and Medical University of Gdansk, Gdańsk, Poland; 3 Department of Biochemical Engineering, School of Chemical Engineering and Technology, Frontier Science Center for Synthetic Biology and Key Laboratory of Systems Bioengineering (MOE), Tianjin University, Tianjin, China

**Keywords:** Bacterial host response, Bacterial infection, Bioinspired materials

## Abstract

*Staphylococcus aureus* (*S. aureus*) can survive inside nonprofessional phagocytes such as keratinocytes, enabling it to evade antibiotics and cause recurrent infections once treatment stops. New antibacterial strategies to eliminate intracellular, multidrug-resistant bacteria are needed. This study used a keratinocyte model infected with methicillin-resistant *S. aureus* (MRSA) to test light-activated compounds, specifically heme-mimetic gallium (III) porphyrin (Ga^3+^CHP) and visible light, known as antimicrobial photodynamic inactivation (aPDI), for eliminating intracellular MRSA. Ga^3+^CHP was found to accumulate more in infected cells, particularly within lysosomal structures where MRSA resides. Flow cytometry and fluorescence microscopy revealed significant colocalization of MRSA and Ga^3+^CHP. Under aPDI, MRSA showed reduced adhesion to host cells and a 70% reduction in the GFP signal from intracellular bacteria. Additionally, light-activated Ga^3+^CHP significantly decreased the number of extracellular bacteria, reducing the potential for further infection. This study is the first to analyze aPDI toxicity in real time within an infection model, demonstrating that this method is neither cytotoxic nor phototoxic.

**Supplementary Information:**

The online version contains supplementary material available at 10.1038/s41598-024-84312-4.

## Introduction

*Staphylococcus aureus*colonizes the skin of approximately 20% of the world’s population and is responsible for 80% of all detected skin infections worldwide^1,2^. Moreover, *S. aureus*plays a key role in the pathogenesis of atopic dermatitis (AD) because its overabundance in the skin microbiota increases inflammation^3^. Staphylococcal infections are difficult to treat because of the production of multiple virulence factors and a high antibiotic resistance profile^4^. Despite antibiotic treatment, 30% of patients are reported to develop recurrent staphylococcal infections after the initial round of antibiotics^5^. Recent scientific reports have shown that one of the defense mechanisms of *S. aureus*against antibiotic action is the invasion of nonprofessional phagocytes such as keratinocytes or fibroblasts^6–8^. *S. aureus*internalization occurs through a complex zipper-like mechanism between fibronectin-binding proteins A and B and fibronectin, which is recognized by α5β1 integrin on host cells^9^. The internalization process differs depending on the bacterial strain and host cell type^6^. *S. aureus*can persist inside host cells for several days (e.g., 4 days) as an intracellular form of bacteria known as small colony variant (SCV) bacteria^10^. This phenotype results in changes in global regulatory networks that can lead to alterations in the production of virulence factors or modify the response to antibiotics^11,12^. When antibiotic pressure is abolished, the intracellular bacterium can escape the endosome and multiply in the cytoplasm. The increased number of bacteria inside the cell leads to cell death, the release of bacteria, and the recurrence of extracellular bacterial infection^13^. Despite the increasing number of studies on *S. aureus*, the mechanism and factors contributing to the entry, survival, and exit of *S. aureus* from the host interior are still not fully understood.

The intracellular phenotype of *S. aureus*is caused and maintained by antibiotic action, as antimicrobials cannot effectively penetrate the cell membrane to achieve efficient intracellular killing^14^. Many new therapies against intracellular *S. aureus*has been proposed^15^. Anti-intracellular bacterial strategies are based on antibiotic modifications to improve their delivery or cell stimulation, which enhances bacterial killing by the host^16–19^. Our study investigated antimicrobial photodynamic inactivation (aPDI) as a potential therapy against intracellular *S. aureus*. Its mechanism is based on three components: a small molecular weight compound with photodynamic properties – a photosensitizer (PS), light at the appropriate wavelength, and an oxygen-rich environment^20,21^. An ideal photosensitizer should exhibit a high reactive oxygen species (ROS) quantum yield with high phototoxicity against pathogens and low toxicity against eukaryotic cells. The penetration of PS into microbial cells should be rapid, with slow uptake in host cells^22,23^. Gallium metalloporphyrins (Ga^3+^MPs) are potent PSs in aPDI that can absorb visible light, such as the green light used in this work^24–26^. Owing to their structural similarity to heme, Ga^3+^MPs, can be recognized by bacterial heme-acquisition receptors of the Isd family and efficiently accumulate inside bacterial cells^27,28^. The porphyrin ring of Ga^3+^MPs is then cleaved inside bacteria and gallium ions are released to disrupt iron-dependent metabolism^24^. We have previously shown that the Ga^3+^MP representative cationic modified gallium porphyrin (Ga^3+^CHP) is water-soluble and exhibits photodynamic activity at Q-band excitation (at lower absorption peaks) with high antimicrobial activity and low toxicity to human keratinocytes^29,30^.

**Fig. 1:**
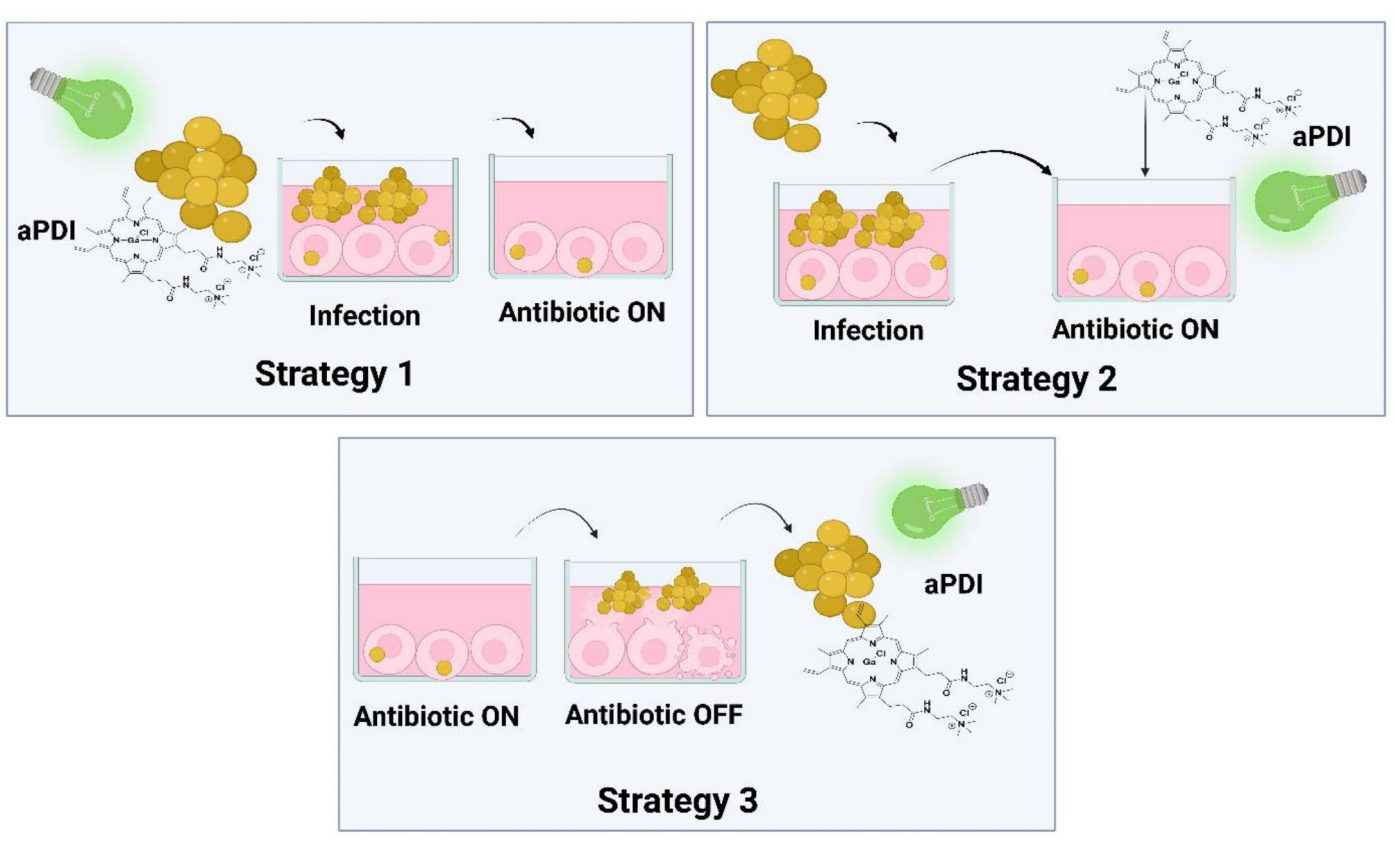
Strategies to implement light-activated gallium metalloporphyrins to overcome *S. aureus* infection of human keratinocytes.

In this study, a human keratinocyte *S. aureus* infection model was constructed and characterized to achieve stable bacterial presence inside host cells. We used this model to investigate whether light-activated Ga^3+^MPs could be applied to effectively inactivate extracellular and intracellular *S. aureus*. In our experimental approach, we used three research strategies based on aPDI action (Fig. 1). Briefly, Strategy 1 involved aPDI treatment of *S. aureus* cells before they contacted and invaded keratinocytes (Fig. 1, Strategy 1). Strategy 2 involved aPDI treatment of intracellular *S. aureus* persisting inside host cells (Fig. 1, Strategy 2). Strategy 3 refers to the aPDI treatment of *S. aureus* that had escaped from an infected cell (Fig. 1, Strategy 3). In the present study, we used two Ga^3+^MP derivatives that we characterized for their antimicrobial efficacy in suspension cells: gallium mesoporphyrin IX (Ga^3+^MPIX)^26^ and Ga^3+^CHP^30^. We demonstrated that the compounds we studied, particularly Ga^3+^CHP, are able to effectively penetrate host cells, localizing mainly to lysosomal structures, where we also detected the presence of *S. aureus*. Using excitation of Ga^3+^CHP with green light, we achieved a significant reduction in the GFP signal originating from intracellular *S. aureus* by aPDI.

Strategy 1 investigated how pretreatment of the bacterial inoculum with aPDI before infection affects *S. aureus* adhesion and internalization in host cells. In Strategy 2, photosensitizers were incubated in the dark to efficiently penetrate and localize inside the host cells to reach the intracellular *S. aureus*. After incubation, the cells were treated with light. Strategy 3 aimed to reduce the potential for recurrent infection by using aPDI on bacteria released from keratinocytes. The antibiotics used in our experimental approach are standard in cell culture.

## Results

### aPDI reduces ***S. aureus*** infection severity and adherence to host cells

**Fig. 2:**
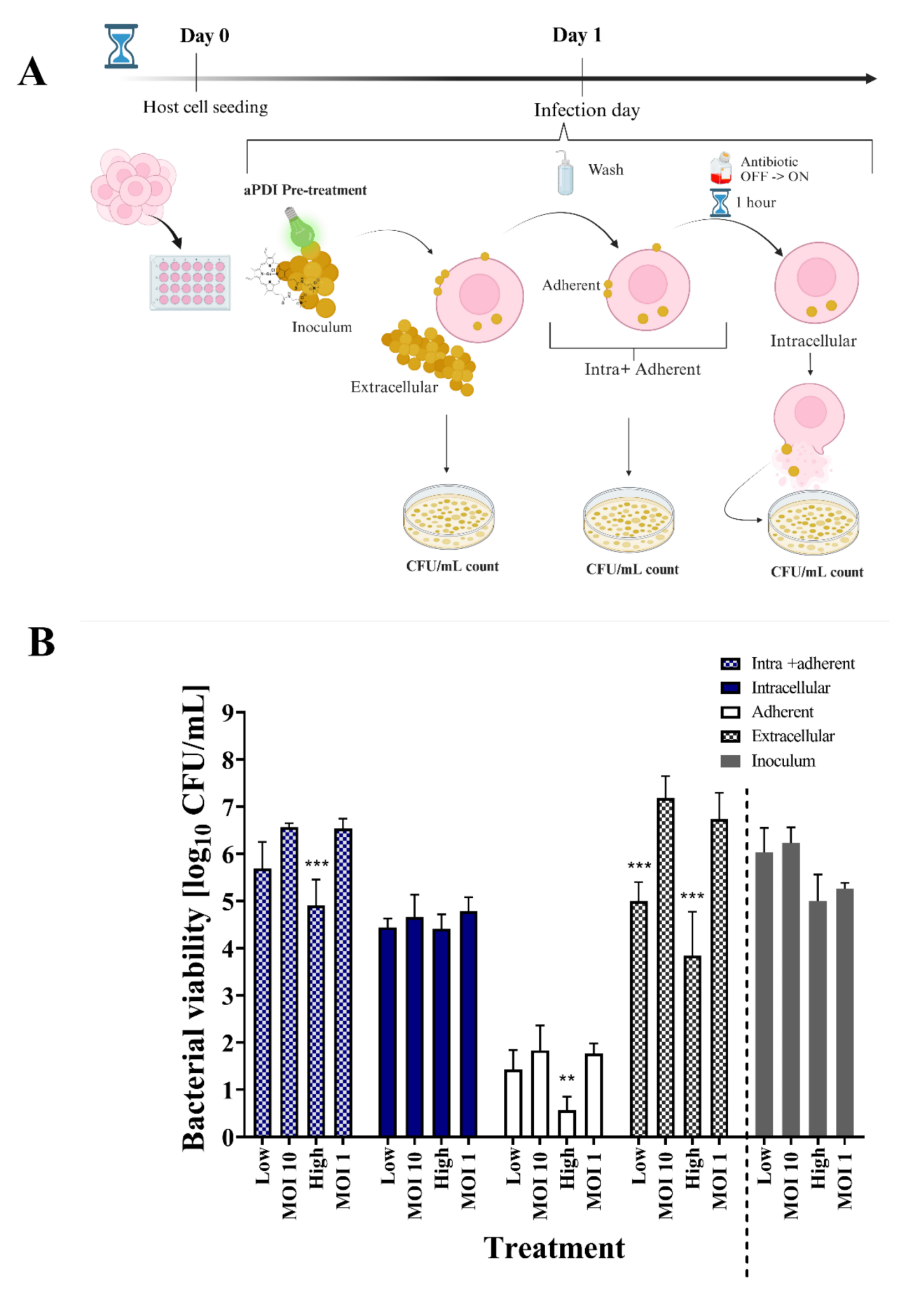
Effect of aPDI (light-activated Ga^3+^CHP) pretreatment on *S. aureus* USA300 infection – Strategy 1. (**A**) On day 0, HaCaT cells were seeded in a 24-well plate. On day 1, the cells were infected with *S. aureus* USA300 that was either untreated or pretreated with aPDI. After 2 h, the growth medium was collected for plating and the extracellular fraction was counted. Host cells were collected for subsequent lysis (intra + adherent fraction) or incubated for 1 h with antibiotics to eliminate adherent bacteria to obtain the intracellular fraction exclusively. All the fractions were serially diluted and plated for CFU/mL enumeration. (**B**) The number of bacteria counted from each fraction collected after infection with an untreated (‘MOI 10’, ‘MOI 1’) or aPDI-treated *S. aureus* inoculum (‘Low’, ‘High’). Before infection, the number of viable *S. aureus* (10^7^ CFU/mL) was reduced by two doses of aPDI: a low dose that resulted in a 1 log_10_ reduction in CFU/mL and high dose that resulted in a 2 log_10_ reduction in CFU/mL. Untreated bacteria with an appropriate MOI of 10 or 1 were used as a control. The number of bacteria used for infection was the same whether the bacteria were pretreated with aPDI or were not pretreated (please see ‘Inoculum’ below). After infection, several fractions were collected, such as ‘Extracellular’ – the free-floating *S. aureus* collected from the medium after infection; ‘Adherent’ - *S. aureus* attached to the host cell; ‘Intracellular’ - *S. aureus* accumulated inside the host cell in the presence of antibiotic pressure; ‘Intra + Adherent’ – the combined number of intracellular and adherent *S. aureus*; and ‘Inoculum’ – the initial number of treated (Low or High) or untreated (MOI 10 or MOI 1) *S. aureus* used for infection. The data are presented as the means ± SDs of six separate experiments. The significant differences in infected cell viability at the respective p values are indicated with asterisks [**p < 0.01; ***p < 0.001], and were normalized to the respective control for each pretreatment (two-way Anova).

Ga^3+^MPs are able to reduce the viability of clinical *S. aureus*isolates in suspension cultures in vitro^29^. In the present study, we wanted to verify whether and at what stage of the infection cycle light-activated Ga^3+^CHP (i.e., aPDI) can be used to reduce the number of bacteria that infect host cells. Therefore we verified 3 strategies for aPDI implementation in infected keratinocytes (Fig. 1). The model of infected keratinocytes was thoroughly characterized and described in the supplementary material (Fig S1 and Fig S2). First, we hypothesized that aPDI pretreatment of the infectious inoculum could affect its further invasion, adherence to the host, and growth of the extracellular bacteria (Fig. 1, Strategy 1). To assess the effect of aPDI pretreatment on the initial stages of *S. aureus* invasion, we studied the number of bacteria in each fraction collected after infection. As a control, we used nontreated infectious inoculum (Fig. 2). Notably, we used the same number of bacteria regardless of the treatment. Furthermore, we wanted to investigate the severity of the infection, so two MOI values, 10 and 1, were used to investigate higher and lower amounts of bacteria. For aPDI-treated bacteria, two doses of aPDI (light combined with 1 µM Ga^3+^CHP) were used, namely, a low dose (2 J/cm^2^) and a high dose (5 J/cm^2^), to reduce the number of bacteria to a value equivalent to an MOI 10 and an MOI 1 (a 1 log_10_ and 2 log_10_ reduction in the number of viable bacteria, respectively). A low dose was used to obtain an MOI of 10 (Fig. 2B, Low), wheras a high dose was used to obtain an MOI of 1 (Fig. 2B, High). After infection, the CFU/mL values were determined in (i) the extracellular fraction from the growth medium after infection, (ii) the intracellular + adherent fraction obtained from a cell lysate containing both intracellular and adherent bacteria, and (iii) the intracellular fraction from a cell lysate where HaCaT cells were washed and cultured under antibiotic pressure for 1 h before lysis to eliminate adherent bacteria (see Fig. 2A). The aPDI-treated bacteria used as an inoculum for infection behaved differently from the untreated bacteria. Compared with no treatment, light-activated Ga^3+^CHP treatment of *S. aureus* resulted in a significantly reduced number of extracellular bacteria, and the observed decrease was 2 log_10_ for the low-dose treatment and 2.8 log_10_ for the high-dose treatment (Fig. 2B). Moreover, high-dose aPDI resulted in a significant reduction in bacterial adherence to HaCaT cells, with a 1.2 log_10_ reduction in CFU/mL count compared with the adherence of the same number of untreated bacteria. Notably, both aPDI-treated bacteria and nontreated bacteria were treated at the same MOI of 1, indicating that the quality but not the quantity of bacteria influenced adherence. Interestingly, pretreatment of bacteria with aPDI did not affect the number of intracellular bacteria, suggesting that there is a maximum bacterial burden that can invade host cells, which is not affected by aPDI. Both aPDI-treated and untreated bacteria penetrated keratinocytes with similar efficiencies. However, aPDI treatment significantly affected the growth of the extracellular fraction and bacterial adhesion to host cells.

### Variable patterns of Ga^3+^ MPs accumulation inside human keratinocytes

**Fig. 3:**
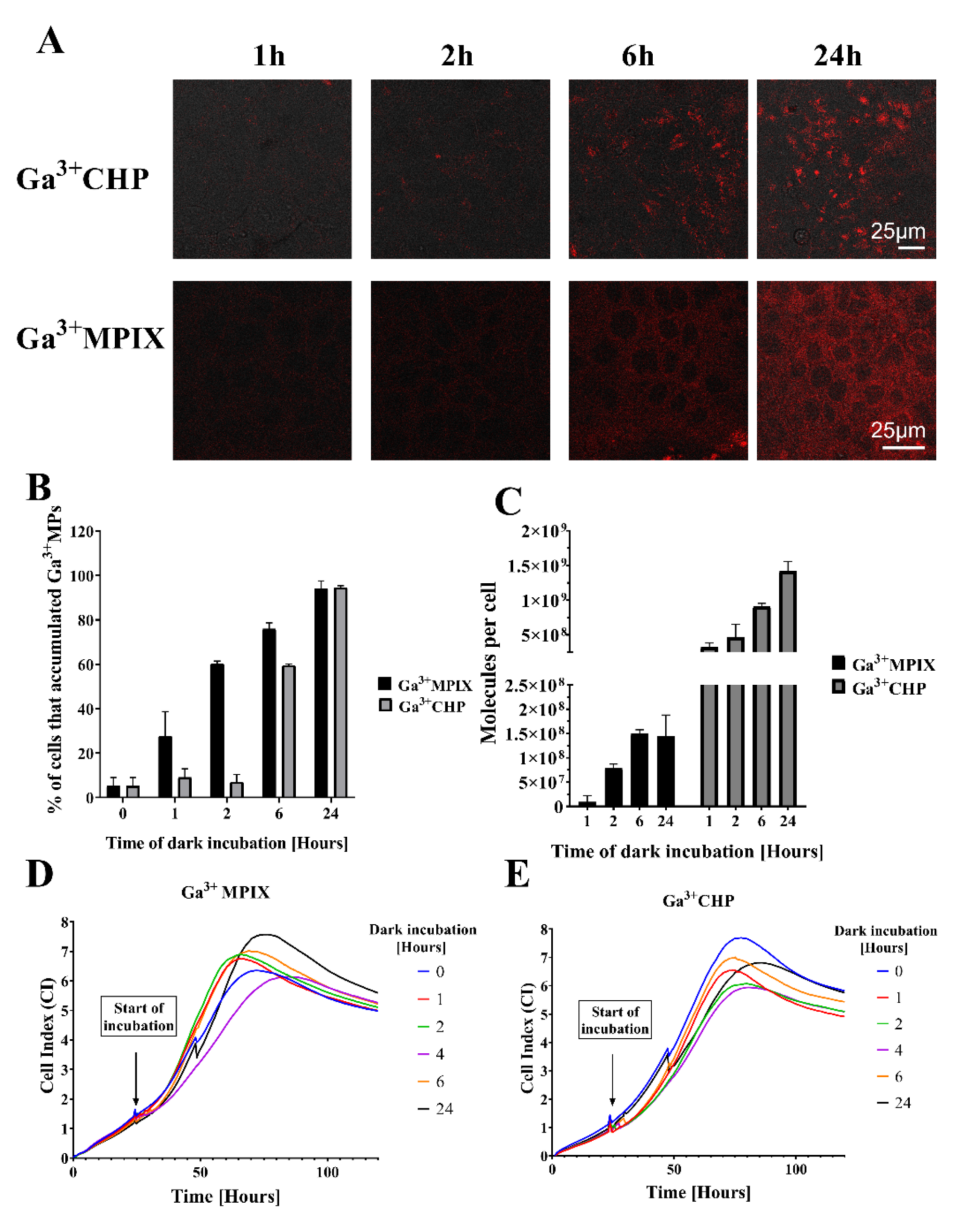
Different accumulation patterns of two gallium metalloporphyrins in human keratinocytes. (**A**) Confocal fluorescence microscopy images showing the accumulation of two gallium compounds, Ga^3+^MPIX and Ga^3+^CHP, in HaCaT cells after incubation in the dark (1–24 h) at a concentration of 10 µM. (**B**) Percentages of cells that accumulated Ga^3+^MPs among the total number of cells measured by flow cytometry. The cells were incubated with 10 µM Ga^3+^MPIX or Ga^3+^CHP and fixed at each time point (1–24 h) in the absence of light, after which the fluorescence signal in the cells was measured by flow cytometry. (**C**) The number of accumulated Ga^3+^MPIX or Ga^3+^CHP molecules (10 µM) in keratinocytes after incubation for the time indicated on the X axis, as measured by the fluorescence intensity of the cell lysate. After incubation in the dark for a particular time, the cells were harvested, counted, and lysed with 0.1 M NaOH/1% SDS to determine the fluorescence of each accumulated compound. (**D**,** E**) Real-time growth analysis of HaCaT cells after incubation in the dark (1–24 h) with 10 µM Ga^3+^MPIX (**D**) or Ga^3+^CHP (**E**).

In the next stage of our work, it was important to carefully examine the accumulation of the tested compounds in host cells before applying Strategy 2 (Fig. 1 Strategy 2), which involves applying aPDI to intracellular bacteria. First, we investigated the accumulation profile of the gallium compounds, Ga^3+^CHP and Ga^3+^MPIX inside human keratinocytes to determine the possible differences and efficacy of the accumulation process. The accumulation of Ga^3+^ MPs is a crucial step for reaching bacteria inside keratinocytes. The rate of accumulation of both compounds was time-dependent. After 1 h of accumulation, both Ga^3+^CHP and Ga^3+^MPIX were localized primarily at the cell membrane (Fig. 3A). However, over time, we observed differences in the localization patterns of the tested gallium derivatives. Ga^3+^MPIX was distributed throughout the cell in the cytoplasm, whereas cationic Ga^3+^CHP was localized mainly inside the cell in clusters (Fig. 3A). Flow cytometry analysis revealed that after 2 h of accumulation, 60% of the keratinocytes accumulated Ga^3+^MPIX, while the corresponding value for Ga^3+^CHP was 7% (Fig. 3B). After 6 h, 75% of the cells accumulated Ga^3+^MPIX and 59% of the cells accumulated Ga^3+^CHP, reducing the differences observed at the 1st and 2nd hours. Interestingly, after 24 h, nearly every cell accumulated both compounds at the same level (94%). When we measured the Ga^3+^CHP or Ga^3+^MPIX accumulation in each cell, we also observed time-dependent uptake (Fig. 3C). Remarkably, despite their accumulation in the cells, none of the tested compounds exerted a significant cytotoxic effect on keratinocytes (Fig. 3D, E). Even after a 24-hour incubation with the test compounds, the cells continued to grow and proliferate until they finally reached the plateau phase at a similar time, regardless of the incubation time with the compound. These results indicated that both compounds accumulate in host cells in a time-dependent manner without significantly affecting host proliferation or viability. However, the accumulation patterns of Ga^3+^MPIX and Ga^3+^CHP inside the cell are highly divergent. Ga^3+^CHP rather than Ga^3+^MPIX, is more likely to reach intracellular *S. aureus* because of its greater localized accumulation inside the cells.

### The accumulation of Ga^3+^ CHP was greater in infected cells

**Fig. 4:**
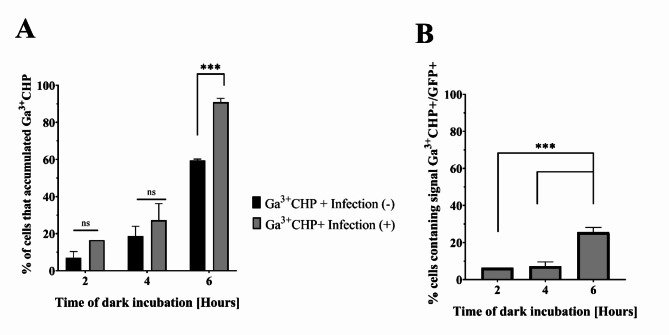
The accumulation of Ga^3+^CHP in infected cells. (**A**) Accumulation of Ga^3+^CHP in infected and uninfected cells. The cells were infected with *S. aureus* USA300 (MOI 10) for 2 h in medium without antibiotics, and then the cells were washed and covered with medium supplemented with antibiotics. Uninfected cells were cultured in parallel. The next day, 10 µM Ga^3+^CHP was added and the cells were incubated in the dark. After incubation, the cells were washed and collected, and the percentage of cells with a fluorescence signal derived from Ga^3+^CHP was determined by flow cytometry. The accumulation results were compared with those of uninfected cells. (**B**) The number of infected cells harboring both GFP + and Ga^3+^CHP + signals after incubation in the dark with Ga^3+^CHP. The significant differences between the two compounds were calculated, and the respective p values are marked with asterisks [***p < 0.001] (one-way Anova).

We next investigated the accumulation of Ga^3+^CHP in *S. aureus*-infected cells. First, keratinocytes were infected with *S. aureus* for 2 h (MOI 10) in medium without antibiotics, and then antibiotics were introduced to remove extracellular bacteria and maintain intracellular invasion. For comparison, uninfected cells were cultured separately. The next day, Ga^3+^CHP was added to the infected cell culture, and the cells were incubated in the dark. After 2, 4, and 6 h of incubation, the cells were collected and the red fluorescence signal from the accumulated compound in both the infected and uninfected cells was detected (Fig. 4A). We observed an increase in the accumulation level over time, measured as the percentage of keratinocytes (infected or noninfected) showing fluorescence produced by Ga^3+^CHP. Interestingly, infected keratinocytes accumulated more Ga^3+^CHP than uninfected cells did. This observation was most evident after a longer incubation (Fig. 4A, 6 h). Next, we analyzed compound accumulation only in the fraction of infected cells containing both signals: red fluorescence from the Ga^3+^CHP compound (Ga^3+^CHP+) and green fluorescence from the *S. aureus* strain USA300 (GFP+) (Fig. 4B). Over time, the accumulation of a gallium compound in the infected cell fraction (Ga^3+^CHP+/GFP+) increased. After 6 h of incubation, the number of infected cells simultaneously exhibiting both signals increased to 25%, indicating that the cationic gallium compound accumulates in infected cells. This finding indicated that cationic Ga^3+^CHP may colocalize with intracellular bacteria.

### Intracellular *S. aureus* colocalizes with Ga^3+^ CHP in keratinocyte lysosomes

**Fig. 5:**
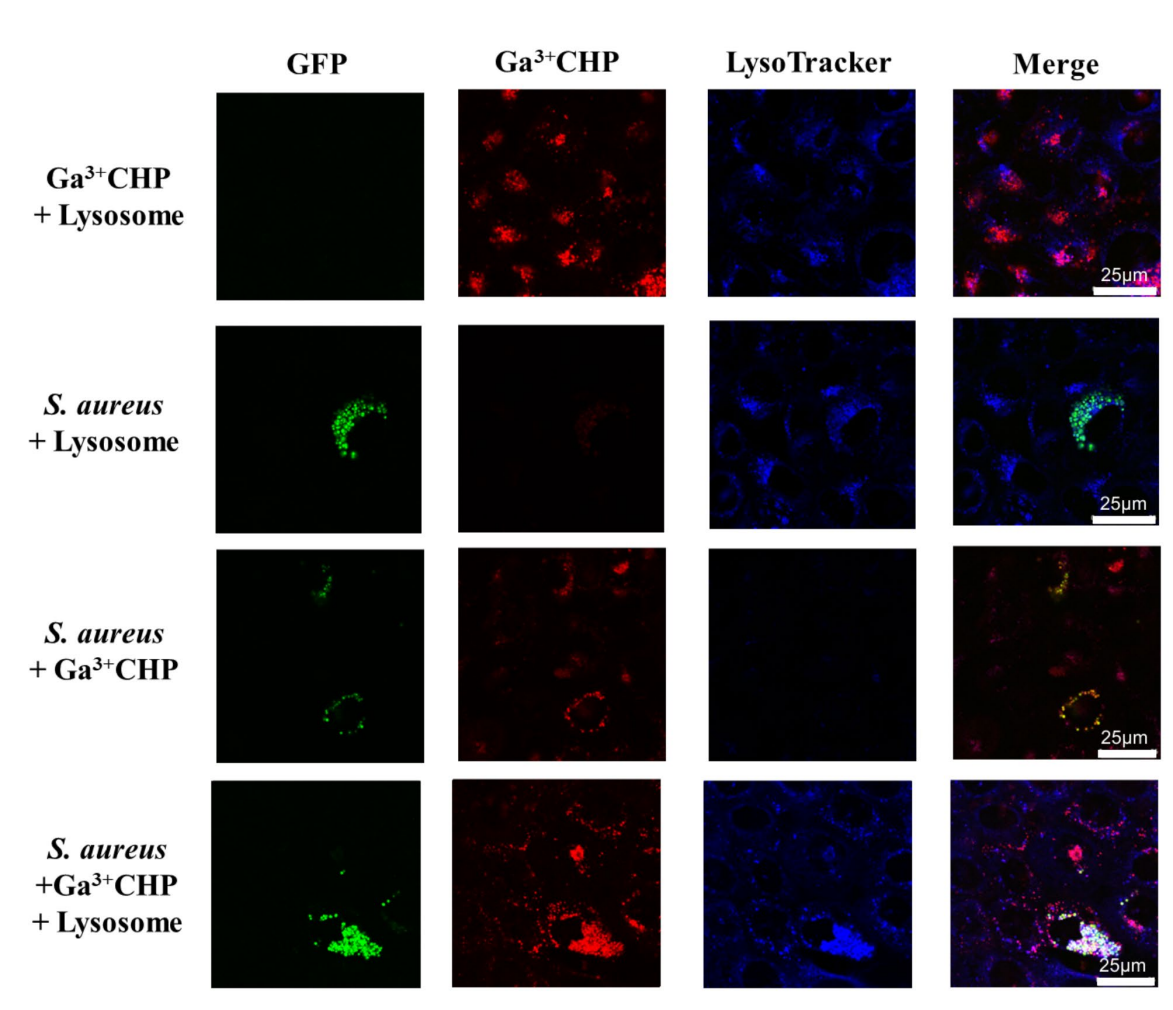
Colocalization of intracellular *S. aureus* and Ga^3+^CHP in lysosomes.

Ga^3+^CHP accumulated inside the cells in distinct clusters; thus, in the next experiment, we wanted to identify the organelles in which the studied compound was localized. Fluorescence dyes specific for the Golgi apparatus, mitochondria, and lysosomes were used to determine the localization of the compound (Fig S5, Table S1). To assess colocalization, images were analyzed for two colocalization coefficients: the Pearson correlation coefficient (significant colocalization > 0.5) and the overlap coefficient (> 0.6). High localization of the compound in lysosomes was observed, but this was not the only site of accumulation. The overlap coefficient between Ga^3+^CHP and LysoTracker™ Deep Red in lysosomes was 0.65 with a Pearson correlation coefficient of 0.46 (Fig. 5), indicating partial localization in lysosomes. We also observed some Ga^3+^CHP accumulation in the mitochondria (although to a far lesser extent than the lysosomes) and no confirmed localization in the Golgi apparatus (Table S1). We subsequently examined the localization of *S. aureus* inside keratinocytes. However, we did not confirm the lysosomal localization of the bacteria in keratinocytes by both the overlap coefficient and Pearson’s correlation (Table S2). Interestingly, when Ga^3+^CHP was incubated with *S. aureus*-infected keratinocytes, colocalization between *S. aureus* and Ga^3+^CHP occurred. Moreover, simultaneous staining of lysosomes and detection of signals from *S. aureus* (GFP) and Ga^3+^CHP indicated the colocalization of these signals in the lysosomes. We thus hypothesized that certain metabolic changes caused by two factors (infection and the presence of Ga^3+^CHP) can promote the colocalization of *S. aureus* inside lysosomes.

Confocal images of *S. aureus*-infected keratinocytes (HaCaT) on the 1st day postinfection, incubated in the dark for 6 h with cationic Ga^3+^CHP (10 µM). The green signal represents intracellular *S. aureus* USA300-GFP, the red signal presents Ga^3+^CHP, and the blue signal represents lysosomes. All colocalization parameters are detailed in Table S2 in the Supporting Information Captions.

### Light-activated Ga^3+^CHP reduces the number of infected keratinocytes

**Fig. 6:**
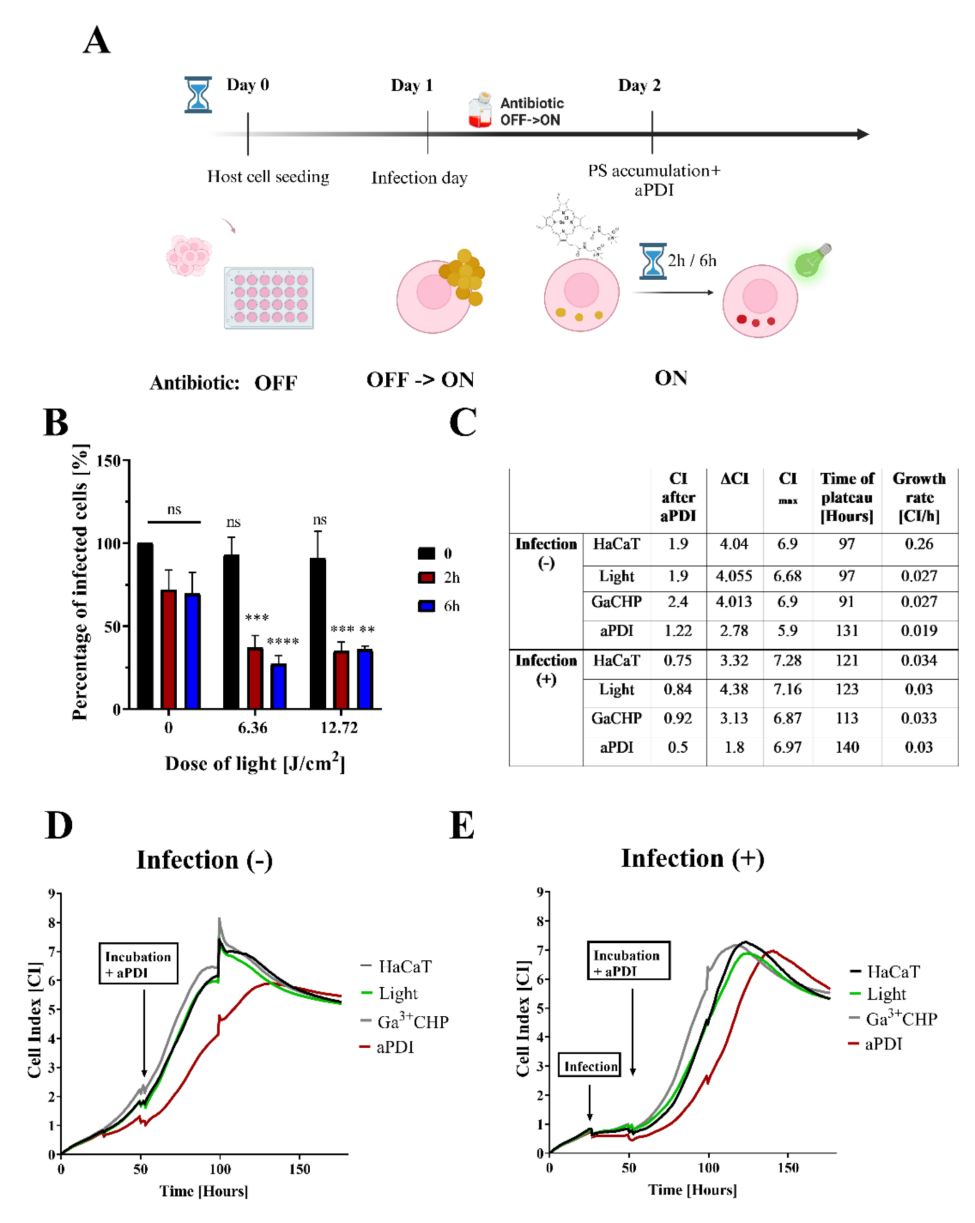
Light-activated Ga^3+^CHP impacts the number of GFP-expressing cells without causing severe host damage. (**A**) Scheme of the experiment. On day 0, HaCaT cells were seeded in a 24-well plate. On day 1, *S. aureus* USA300 (MOI 10) was added, and the cells were incubated for 2 hours. Next, the medium was replaced with antibiotic medium (Antibiotic OFF-> ON) to eliminate extracellular bacteria and maintain intracellular invasion. On day 2, Ga^3+^CHP was added to the medium and incubated for 2–6 h in the dark. Then, the cells were washed, and green light was applied at the proper dose. (**B**) Percentage of infected cells after aPDI. The number of GFP-expressing cells after 2- or 6-hour incubation in the dark followed by green light illumination. The cells were collected and fixed, and the GFP signal was measured by flow cytometry. The results were calculated in reference to the untreated control (cells with no compound and no light exposure) (two-way Anova). (**C**) Table showing the growth characteristics of infected or uninfected cells subjected to different treatments. The following parameters were accounted for in the analysis: the cell index (CI) immediately after aPDI treatment (at the 55th hour of the experiment); the ΔCI which is characterized by the growth rate immediately after aPDI treatment, was calculated as the difference in the growth of cells after aPDI treatment (from 55 h of the experiment) and cells in the middle of logarithmic growth (at ~ 100 h of the experiment); the CI_max_- maximum CI achieved at the beginning of the plateau phase; the time to plateau phase - time at which cells enter stationary growth phase; and the growth rate, which was calculated as the total rate of increase in the logarithmic phase of growth after treatment to the time at which the plateau phase of the analysis curve was reached. (**D**, **E**) Real-time growth analysis of HaCaT cells that were not infected (**D**) or infected (**E**) after incubation in the dark for 2 hours with 10 µM Ga^3+^CHP and with or without light illumination of 6.36 J/cm^2^. The CI was measured by a real-time cell analysis (RTCA) instrument every 10 min. The experiments were conducted until the cells reached the plateau growth phase.

After establishing the colocalization of intracellular bacteria and Ga^3+^CHP, we analyzed the effects of aPDI on intracellular and intralysosomal *S. aureus* according to Strategy 2 (Fig. 1 Strategy 2). To this end, we excited the accumulated Ga^3+^CHP in the cells with green light and investigated how it affected the total number of infected cells. For this purpose, on the first day, we infected keratinocytes, removed extracellular bacteria, and left only intracellular bacteria by adding antibiotics to the culture. The next day, we added Ga^3+^CHP to the cells and incubated them for 2–6 h in the dark. After incubation, we irradiated the cells with green light at doses of 6.36–12.72 J/cm^2^ (Fig. 6A). Using flow cytometry, we determined the number of infected cells by tracing the GFP signal originating from intracellular *S. aureus*. As a control (100%), the GFP signal of infected cells that were not subjected to aPDI was analyzed. The incubation of the cells with Ga^3+^CHP in the dark reduced the number of cells expressing GFP to ~ 70%, regardless of the incubation time. However, when green light irradiation was added, a significant decrease in the number of GFP-expressing cells to 37 − 27% was observed, which was not dependent on incubation time or the light dose applied (Fig. 6B). Treatment with light alone did not reduce the number of infected cells. For Ga^3+^MPIX, which did not accumulate in intracellular clusters, we did not observe any decrease in the number of infected cells after green light illumination (Fig S6). The colocalization of both the photosensitizer and the bacteria is crucial for the effective reduction of intracellular *S. aureus* during the aPDI process.

To verify how aPDI treatment affects the survival and growth of host cells, we analyzed the real-time growth of infected and noninfected cells after illumination with green light (6.36 J/cm^2^) following a 2-hour incubation with Ga^3+^CHP in the dark (Fig. 6C-E). We observed that incubation of cells with Ga^3+^CHP followed by light application delayed the proliferation rate of both infected (Fig. 6E) and uninfected (Fig. 6D) cells. Interestingly, after aPDI, infected cells presented a greater overall growth rate (0.03 CI/h) than uninfected cells did (0.019 CI/h) until they reached the plateau phase. However, cell growth resumed immediately after aPDI in uninfected cells compared with infected cells (as reflected by the ΔCI parameter: 2.78 vs. 1.8 for noninfected vs. infected cells, respectively). The plateau phase was reached faster in uninfected cells (131 h, CI_max_ = 5.9) than in infected cells (140 h, CI_max_ = 6.9). Compared with untreated cells, both infected and uninfected cells presented significant reductions in growth and a delays in entering the plateau phase after aPDI treatment. Despite some phototoxicity from aPDI, which reduced the rate of cell proliferation after treatment, both infected and uninfected cells overcame photodamage and began to proliferate until the plateau phase was reached. This finding indicated a lack of significant photo- and cytotoxicity against host cells.

### Green light-activated Ga^3+^MPs efficiently eliminate recurrent staphylococcal infection

**Fig. 7:**
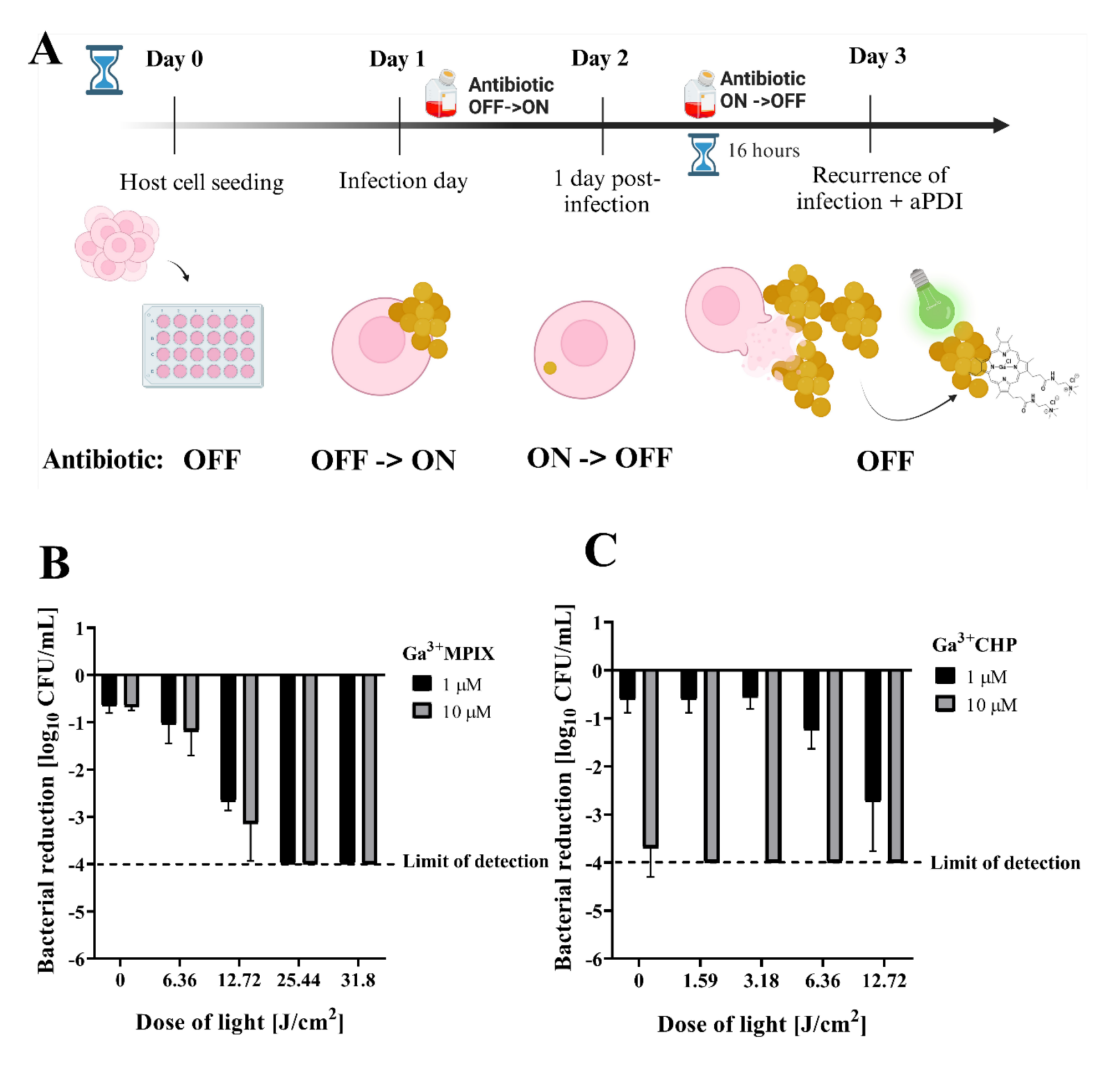
Light-activated Ga^3+^MPs effectively reduce the number of *S. aureus* USA300 released from keratinocytes – Strategy 1. (**A**) On day 1, HaCaT cells were infected with *S. aureus* USA300 (MOI = 10, 2 h) in medium without antibiotics (Antibiotic OFF). An antibiotic was added to remove extracellular *S. aureus*, and the cells were cultured under antibiotic pressure until the next day (Antibiotic OFF-> ON). The antibiotic was then removed (Antibiotic ON-> OFF), and HaCaT cells containing only intracellular *S. aureus* were left in an incubator for 16 h. During this time, intracellular *S. aureus* gradually lysed the cells and was released into the medium. *S. aureus* cells were harvested, washed with DMEM and then resuspended in tryptic soy broth (TSB). The bacterial suspensions were transferred to a 24-well plate, proper photosensitizers were added, and after incubation, they were illuminated with green light. The bacterial cells were then diluted, plated, and counted (**B** and **C**). The reduction in the number of *S. aureus* USA300 bacteria caused by light activation of Ga^3+^CHP (**B**) or Ga^3+^MPIX (**C**) was calculated in relation to that in the untreated cells.

On the basis of the third proposed aPDI strategy (Fig. 1, Strategy 3), we evaluated the antimicrobial efficacy of Ga^3+^MPs activated with 522 nm light against *S. aureus* USA300 in staphylococcal reinfections. Briefly, the cells were seeded on day 0, *S. aureus* infection was performed on day 1, and then the cells were cultivated in medium supplemented with an antibiotic (Antibiotic ON) (Fig. 7A). On day 2, the culture medium was changed to antibiotic-free medium (Antibiotic OFF), and the cells were cultivated for up to 16 h. Extracellular bacteria that were released from keratinocytes were isolated, washed, and subjected to aPDI using two Ga^3+^MPs, Ga^3+^MPIX or Ga^3+^CHP, to assess their efficacy in reducing *S. aureus* survival (Fig. 7B and C). Both gallium compounds effectively eliminated bacteria released from cells with a maximum reduction in the cell count of 4 log_10_ CFU/mL. Ga^3+^CHP was more effective than Ga^3+^MPIX in reducing bacterial survival at lower light doses, corresponding to shorter irradiation times. We found that *S. aureus*, which was released by the host and restarted the infection, responded to aPDI like bacteria in the logarithmic growth phase rather than the stationary phase (Fig S4). Despite some adaptive changes in intracellular bacteria that might promote their overall tolerance, the released *S. aureus* showed a similar response to aPDI to that of bacteria in free suspension cultures during the logarithmic growth phase (Fig. S4). These finding demonstrated that aPDI with Ga^3+^MPs can be an effective strategy to combat staphylococcal reinfections.

## Discussion

In patients with chronic and recurrent skin infections, such as AD, preventing and treating staphylococcal infections are extremely important. The interactions between *S. aureus* and skin cells are the subject of intensive research. On the one hand, investigations on whether there are specific characteristics that allow bacteria to adapt to living on the skin are being conducted. On the other hand, host cells, which are not just passive players in the host‒pathogen interaction, are the focus of research. Methods are constantly being developed to control the spread of staphylococci on the skin, which occurs frequently in individuals with skin diseases, such as AD. Antibiotics are used to combat staphylococcal skin infection, but the frequent use of antibiotics leads to the selection of strains resistant to the drug used. In this case, other methods are needed, preferably those that can combat infections caused by drug-resistant bacteria. One method that is described in this article is aPDI. It is a method with proven efficacy against not only *S. aureus*but also other microorganisms, including drug-resistant pathogens^21^. However, it has not yet been shown whether the use of aPDI is effective against intracellular bacteria.

Recent scientific reports indicate the ability of *Staphylococcus aureus*to invade nonprofessional phagocytes, including keratinocytes. In this way, bacteria avoid the effects of antibiotics, which cannot penetrate the host cell membrane^6,31^. In our study, we established and characterized a keratinocyte infection model (Fig S1 and Fig S2) and investigated the efficacy of aPDI in treating multidrug-resistant *S. aureus* at different infection stages: adherence, intracellular persistence, or release of bacteria from the host cell (Fig. 1).

Once internalized, *S. aureus*is exposed to two selective pressures: antibiotics and the intracellular environment of the host. The pathogen undergoes drastic transcriptional changes to increase its persistence^32,33^. Despite these adaptive changes, intracellular bacteria still exhibit metabolic activity. Moreover, it has been shown that the intracellular phenotype could manifest greater tolerance to antibiotics^34^. The reservoir of intracellular *S. aureus*is strongly linked to the recurrence of infection^35–37^. Under favorable conditions (i.e., the absence of antibiotics), *S. aureus* USA300 could escape the cells and reinduce the infection, even with a lower intracellular load (Fig S1, Fig S3). The same observation was made by Rollin et al., where *S. aureus*resumed extracellular infection after maintaining the intracellular inoculum for up to 10 days under selective antibiotic pressure^38^. Bacteria that are released from the host might contribute to therapeutic failure. In our study, we evaluated the efficacy of aPDI (Strategy 3) in eliminating *S. aureus* released from host cells that had regrown from the intracellular inoculum. Our results indicated that bacteria released from cells were still sensitive to aPDI, similar to suspension cultures of *S. aureus* grown in vitro (Fig. 7). The adaptive changes that occurred during infection did not alter bacterial sensitivity to aPDI. These findings indicate that the combination of green light and Ga^3+^MPs might be an efficient strategy to combat recurrent staphylococcal infections.

In our previous study, light-activated Ga^3+^CHP was shown to be an efficient singlet oxygen producer^29^. The singlet oxygen generated during aPDI pretreatment might affect the structure of bacterial surface proteins responsible for their attachment to the host^39^. Moreover, a previous study showed that aPDI reduces biofilm adherence to abiotic surfaces^40^. Our results showed for the first time that aPDI using green light-activated Ga^3+^CHP reduced the adhesion of *S. aureus* to a biotic surface, namely, keratinocytes (Fig. 2). Interestingly, pretreatment of bacteria with aPDI before infection (Strategy 1) did not significantly change the level of internalization of *S. aureus*, regardless of inoculum size (MOI 10 vs. MOI 1) or aPDI strength (High vs. Low), and the number of intracellular *S. aureus* bacteria did not change (Fig. 2). These results indicate that internalization is prioritized by *S. aureus* during infection until a certain capacity for host internalization is achieved.

Some challenges need to be overcome for aPDI to be used as an anti-intracellular therapy^41,45^. The ideal scenario is killing infected keratinocytes while sparing noninfected keratinocytes. To achieve this selective killing, the PS must accumulate inside the host cells and perhaps inside the intracellular vesicles where the pathogen may reside, which is a major challenge. For this reason, investigating the intracellular localization of both the pathogen and the PS is key to verifying the potential of aPDI treatment for treating intracellular pathogens. The second important issue is the effective cellular concentration of PS, which depends not only on the accumulation process but also on the potential efflux of PS or the unwanted interaction of PS with host cell biomolecules. We studied Ga^3+^MPs and revealed divergent cellular localization patterns in keratinocytes. In general, the accumulation of porphyrins in eukaryotic cells occurs through a slow passive diffusion process with partial accumulation within mitochondria^41,42^. In our study, cationic Ga^3+^CHP was mostly localized in lysosomal structures (Fig. 5) and partially localized in mitochondria (Fig. S5 B). The presence of cationic quaternary ammonium moieties in the structure of Ga^3+^CHP might increase its targeting and uptake by lysosomes through electrostatic attraction^43,44^. At this stage of research, it is difficult to determine whether Ga^3+^CHP first accumulates in lysosomes, attracting bacteria to these structures, or whether bacteria capture Ga^3+^CHP via highly specialized heme import systems, consequently ending up in lysosomes. On the one hand, the environment of the lysosome is nutrient-poor, which favors the bacterial cell changing its phenotype to a dormant one and thus becoming more resistant to antimicrobials. On the other hand, in poor environments, pathogens such as *S. aureus* produce highly specialized proteins that efficiently capture nutrients such as heme or its structural analogs, as demonstrated in this work and our previous studies (Fig. 4)^26,29^. Interestingly, we did not confirm the lysosomal localization of intracellular *S. aureus* itself (Fig. 5; *S. aureus* + Lysosome). However, simultaneous colocalization of intracellular bacteria with Ga^3+^CHP in the lysosomal structures of the host was confirmed (Fig. 5; *S. aureus* + Ga^3+^CHP + Lysosome). The presence of both *S. aureus* and Ga^3+^CHP may influence metabolic changes that favor the colocalization of *S. aureus* inside lysosomes. Therefore, there is a high probability that Ga^3+^CHP can reach intracellular bacteria or even accumulate intracellularly through heme receptor acquisition systems as a part of the light-independent action of Ga^3+^MPs.

With Strategy 3 presented in this study, we tested the effectiveness of aPDI on the intracellular load of *S. aureus* in keratinocytes. To date, few studies have investigated the effectiveness of aPDI in eliminating intracellular bacteria. For example, efficient aPDI killing of intracellular *S. aureus*in HeLa cells was achieved with red light and a conjugate of the cell-binding domain of endolysin (CBD3) and silicon phthalocyanine (700DX)^45^. Furthermore, blue light-activated gallium-substituted hemoglobin loaded on silver nanoparticles was used against intracellular *S. aureus*, which persisted inside professional phagocytes^46^. Both examples use high-molecular-weight bioconjugates, which may have more difficulty accumulating inside eukaryotic cells than low-molecular-weight compounds do. Previous studies on aPDI efficacy in this field have focused on infection models involving either cancer cell lines or professional phagocytes, such as macrophages^45,46^. Our study is the first evaluation of the efficacy of aPDI in eliminating *S. aureus*that reside in keratinocytes. The accumulation of bioconjugates may be difficult for nonprofessional phagocytes because of the lower accumulation capacity of these cells^47^. We used small-molecular-weight compounds, gallium metalloporphyrins, rather than bioconjugating with large molecules. The addition of a positive charge gained by quaternary ammonium moieties increases the hydrophilicity of the compound, which might be important for PS lysosomal localization inside eukaryotic cells^48^. Similar results were observed for the intracellular localization of hydrophilic sulfonated tetraphenyl porphyrins^49^. Moreover, adding a cationic charge has been reported to be necessary for effective photokilling and increased accumulation of benzophenoxazine in the intracellular pathogen *Leishmania*^50^. We previously reported that Ga^3+^CHP is a biocompatible and safe agent both in vitro and in vivo^30^. Green light excitation of Ga^3+^CHP did not exhibit pronounced phototoxicity in studies on HaCaT cell cultures alone^29^ or in our infectious model presented in this work (Fig. 6). This makes Ga^3+^CHP combined with green light a promising anti-intracellular treatment that has a low phototoxicity impact on keratinocytes despite their infection status. One limitation of this study is the inability to distinguish whether aPDI causes elimination of only intracellular bacteria without damaging keratinocytes or bacteria together with the keratinocytes that incorporate them.

Overall, this research presents the application potential of light-activated compounds for (i) the elimination of staphylococcal recurrent infections, (ii) a reduction in infection severity and bacterial attachment to host cells, and (iii) the significant elimination of intracellular *S. aureus*. We highlighted the great importance of the simultaneous colocalization of a PS and intracellular bacteria within host cells to effectively reduce the number of infected cells by aPDI.

## Materials and methods

### Bacterial strains and growth conditions

In this study, we used two GFP-expressing *S. aureus*strains: hypervirulent USA300 (AH3369) derived from A. Horswill^51^. Strain was grown in trypticase soy broth (TSB, bioMérieux, France) at 37 °C with 10 µg/mL of chloramphenicol for USA300. To obtain bacteria in the stationary phase of growth, overnight cultures were grown for 16–20 h and diluted to 0.5 McFarland (~ 10^7^ CFU/mL). For infection, stationary overnight cultures were diluted to 1:100 in flask and grown with shaking (150 rpm/37°C) to obtain logarithmic phase up to 2 h then diluted to 0.5 McFarland for further investigations.

### Cell line and culture media (antibiotic ON/OFF)

The human immortalized keratinocyte HaCaT (DKFZ, Heidelberg, Germany) cell line was used in this study. Culture medium with antibiotic (Antibiotic ON) consisted of Dulbecco’s modified Eagle’s medium (DMEM) with 10% fetal bovine serum (FBS), 4.5 g/L glucose, 1 mM sodium pyruvate, 100 U/mL penicillin, 100 µg/mL streptomycin, 2 mM l-glutamine, and 1 mM nonessential amino acids. The medium without antibiotic (Antibiotic OFF) was based on the previously given composition of DMEM HaCaT growth medium but without the addition of streptomycin and penicillin. All media components were derived from Gibco, Thermo Fisher Scientific, USA. Cells were grown in a standard humidified incubator at 37 °C in a 5% CO_2_ atmosphere.

### Co-culture methodology

To obtain intracellular *S. aureus*, we proposed the infection model protocol, Briefly, HaCaT cells were seeded in the antibiotic-free medium (Antibiotic OFF) prior to infection day, referred to as Day 0, at a density of 10^5^ cells/per well in a 24-well plate (Corning, USA). The next day (Day 1), overnight *S. aureus* cultures were diluted 1:100 in the 10 mL of TSB with proper antibiotic to sustain the plasmid in the flask. Cells were grown for 2 h with 150 rpm shaking at 37 °C to achieve logarithmic growth. Bacterial suspensions were diluted in TSB to 0.5 McFarland (~ 10^7^ CFU/mL), and depending on the MOI for infection, the appropriate concentration of bacteria was added to the growth medium of HaCaT cells. After 2 h of infection incubator at 37 °C in a 5% CO_2_ atmosphere, cells were washed with PBS, and the medium was changed to contain the standard cultures antibiotics (Antibiotic ON). During the following days (Day 2–7), either the antibiotic pressure was maintained, or the medium was changed for non-antibiotic (OFF) to release bacteria from the host.

To investigate the *S. aureus* fraction to count CFU/mL, we collected each fraction at the proper time point of the infection model preparation. For extracellular *S. aureus*, the fraction was collected from the growth medium after 2 h of infection. For the intra + adherent fraction, after infection, cells were washed three times with PBS, and trypsinized with TrypLE™ Express (Thermofisher Scientific, USA). Cells were collected, washed, centrifuged, then resuspended in the 0.1% TritonX-100 in MiliQ for 30 min for cell lysis. For intracellular *S. aureus*, 2 h after the addition of *S. aureus* infectious inoculum, cells were washed, and the growth medium was change to fresh medium with antibiotic (Antibiotic ON) to kill the adherent and extracellular bacteria. After 1 h incubation with antibiotics, the cells were washed and harvested. After that, samples were washed and lysed with lysis buffer. Each fraction was serially diluted and placed on TSA agar plate for CFU/mL counting.

To prepare the fraction of *S. aureus* from different stages of infection for analysis on the flow cytometer. After infection, cells were washed with PBS, then trypsinized and harvested. Culture medium was used to neutralize the trypsin, and the cells were washed with PBS and resuspended in BD Cytofix/CytoPrem™ kit (BD Biosciences, USA). After a 20-minute incubation in the dark with pre-fix buffer, the cells were centrifuged, washed twice, and resuspended in PBS for further analysis on flow cytometer.

On the Day 0, HaCaT cells were seeded in a 24-well plate in a non-antibiotic medium (Antibiotic OFF). On day 1, *S. aureus* overnight cultures were diluted and grown by 2 h to achieve a logarithmic growth phase. Afterward, cultures were diluted and transferred to the cells at a proper multiplicity of infection. Then, after 2 h of infection, the growth medium was changed to antibiotic (Antibiotic ON) to put antibiotic pressure on intracellular invasion. On days 2–7, the antibiotic pressure was maintained or lifted by changing the medium to non-antibiotic (Antibiotic OFF) to release extracellularly intracellular *S. aureus*.

### Gallium compounds

The synthesis and structure characterization of cationic gallium porphyrin (Ga^3+^CHP) and Ga^3+^ mesoporphyrin IX chloride (Ga^3+^MPIX) was previously described in detail^29,30^. The initial stock of Ga^3+^CHP was prepared in Milli-Q water, while Ga^3+^MPIX in the 0.1 M NaOH solution.

### Compound accumulation

The intracellular accumulation of each photosensitizer inside human keratinocytes was analyzed by two methods: flow cytometer analysis and measurements of fluorescence intensity of cell lysates. Ga^3+^MPIX or Ga^3+^CHP at 10 µM were added to the medium of HaCaT cells either infected or uninfected. After dark incubation at the desired time, the external photosensitizer was removed, then cells were trypsinized and collected. Cells were either fixed with BD Cytofix/CytoPrem™ kit for flow cytometry analysis or counted and then lysed with 0.1 M NaOH/1%SDS buffer for fluorescence lysate measurements. The fluorescence intensity of each sample was measured with an EnVision Multilabel Plate Reader (PerkinElmer, USA) at the following emission/excitation wavelengths: Ga^3+^MPIX at 406/573 nm and Ga^3+^CHP at 406/582 nm. Accumulation calculations for each PS were made from a compound calibration curve prepared in the lysis solution. The uptake values are presented as PS molecules accumulated per HaCaT cell number in the well. The molecular weight of Ga^3+^CHP was calculated to be 907.08 g/mol, and that of Ga^3+^MPIX was estimated to be 669.85 g/mol.

### Flow cytometry

Prepared fixed cells were analyzed by GFP signal originating from *S. aureus* that was detected with the green detector channel (exc 488 nm, ems 525/30 nm). For the accumulation of photosensitizer - the PS signal detected the Red Detector channel (exc 642 nm, ems 664/20 nm). Cells were analyzed using Guava easyCyte™ flow cytometer and guavaSoft 3.1.1. software with an analysis of 10,000 events.

### Light activation of Ga3+MPs (strategy 1, 2 and 3)

For the light-activation of gallium compounds, we used a light-emitting diode (LED) emitting green 522 nm-light (irradiance = 10.6 mW/cm^2^, FWHM = 34 nm) to excitement either Ga^3+^MPIX or Ga^3+^CHP. The emission spectra of LED was previously characterized in our previous research^52^.

**Strategy 1**: the overnight *S. aureus* was diluted to 0.5 McFarland (10^7^ CFU/mL), then 900 µL of bacterial aliquots were placed on the 24-well plates with 100 µL of 10x concentrated photosensitizer, followed by 10 min dark incubation with shaking at 37 °C. Then, samples were illuminated. For a low dose of aPDI, 1 µM of Ga^3+^CHP was used with 2 J/cm^2^ dose of green light (1 log_10_ of reduction in bacterial survival, final bacterial concentration of ~ 10^6^ CFU/mL), while for a high dose, we used 1 µM of Ga^3+^CHP and 5 J/cm^2^ (2 log_10_ of reduction, final bacterial concentration: ~10^5^ CFU/mL). Samples were centrifuged (10 000 rpm/5 min), then resuspended in 100 µL of fresh TSB before addition to the 10^5^ HaCaT cells to start the infection. Untreated cells were added at the proper MOI (10 or 1) as a corresponding control.

**Strategy 2**: either Ga^3+^MPIX or Ga^3+^CHP at 10 µM concentration was added to infected cells at the 1st day post-infection. Then samples were left for dark incubation in the CO_2_ incubator for 2–6 h. The PS-containing medium was replaced with PS-free medium for green light illumination at dose of either 6.36 J/cm^2^ or 12.72 J/cm^2^. Afterwards, cells were prepared for flow cytometry analysis. The same protocol was conducted for real-time growth analysis of the cells on the xCELLigence device, however, after illumination, cells were left for measurements of the Cell Index (CI) parameter.

**Strategy 3**: HaCaT cells were infected with *S. aureus* at MOI 10 for 2 h in an antibiotic-free medium (Antibiotic OFF). Then, cells were washed with PBS, and the medium was changed to antibiotic (Antibiotic ON). The next day, the medium was removed, adherent cells were washed with PBS, then the antibiotic-free medium was again applied (Antibiotic OFF) and cultivated for 16–20 h to release the intracellular *S. aureus* from the host. Then, the medium was collected and centrifuged (10 000 rpm, 5 min), and bacterial sediment was resuspended in 1 mL of fresh TSB. The 10 µL of the bacterial sample was taken to count the number of bacteria released from the co-culture. The bacterial aliquot of 450 µL was mixed with 50 µL of 10x concentrated PS, then incubated in the dark with shaking at 37 °C. The light illumination was applied, and surviving bacteria were serially diluted and placed on the TSA agar plates for CFU/mL counting.

### Real-time growth analysis

For real-time analysis of cell growth dynamics, HaCaT cells were seeded the day before the experiment at a density of 10^4^per well on an E-plate (ACEA Biosciences Inc., USA) and placed in the xCELLigence real-time cell analysis (RTCA) device (ACEA Biosciences Inc., USA). The next day, cells were treated according to the purpose of experiemtn; either infection with proper MOI was conducted, or photosensitizer was added to the dark incubation studies. The Cell Index (CI) reflected the change in the flow of electrons between electrodes on the E-plate. The value of impendence depends on the cell type, the density, its shape, and the degree of adhesion to the well^53^. The CI parameter was monitored every 10 min until the plateau phase of the cell growth was reached.

### Confocal imaging

Specimens were imaged using a confocal laser scanning microscope (Leica SP8X equipped with an incubation chamber for the live analysis) with a 63× oil immersion lens (Leica, Germany). Cell nucleus for intracellular persistence studies and 3D-dimension images was stained with HOEST 33,342 dye (Thermofisher Scientific, USA). For lysosomal staining the LysoTracker™ Deep Red (Sigma-Aldrich, USA) was used; for mitochondria – Mito RED (Sigma-Aldrich, USA); for the Golgi apparatus – GOLGI tracker NBD c6 ceramide (Thermofisher Scientific, USA). Pixel intensities were quantified and evaluated using the Pearson’s correlation or the overlap coefficient to derive the colocalization rate (%). Quantitative analyses of colocalizations were performed using Leica Application Suite X, version 3.5.2.18963.

### Statistics

Statistical analysis was performed using GraphPad Prism 8 (GraphPad Software, Inc., CA, USA). Quantitative variables were characterized by the arithmetic mean and the standard deviation of the mean. Data were analyzed using one-way or two-way ANOVA with Dunnett’s multiple comparison test.

## Electronic supplementary material

Below is the link to the electronic supplementary material.

Supplementary Material 1

Supplementary Material 2

Supplementary Material 3

Supplementary Material 4

Supplementary Material 5

Supplementary Material 6

Supplementary Material 7

## Data Availability

The datasets generated during the current study are available upon request from the corresponding author Joanna Nakonieczna.
