# Supplementary figures and images for: Harnessing light-activated gallium porphyrins to combat intracellular Staphylococcus aureus using an in vitro keratinocyte infection model
Source: Sci Rep. 2025 Jan 8;15:1295. doi: 10.1038/s41598-024-84312-4 (PMC11711192; doi:10.1038/s41598-024-84312-4)

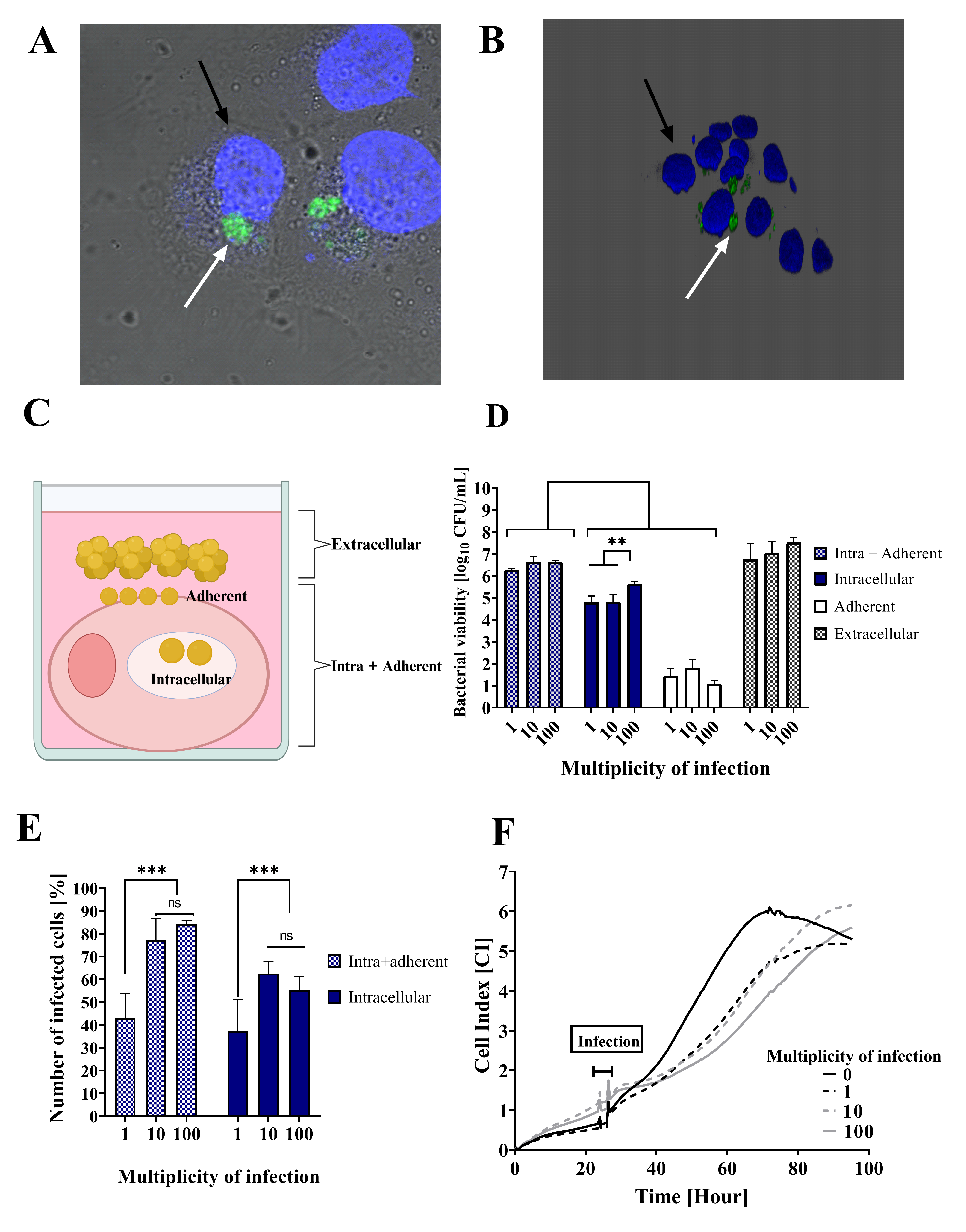

Supplement: Supplementary file 1 — Supplementary Material 1 [file 41598_2024_84312_MOESM1_ESM.tif]

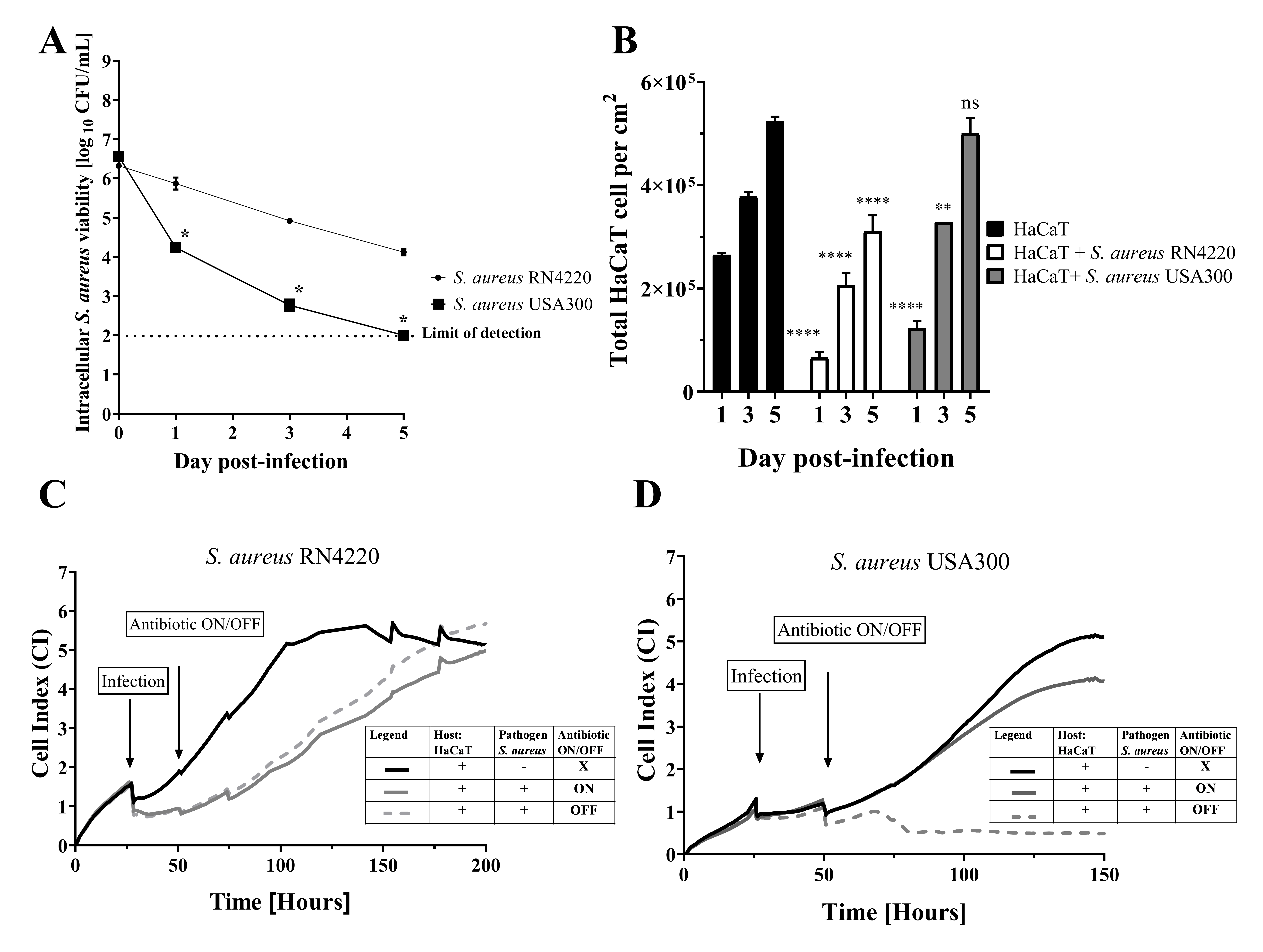

Supplement: Supplementary file 2 — Supplementary Material 2 [file 41598_2024_84312_MOESM2_ESM.tif]

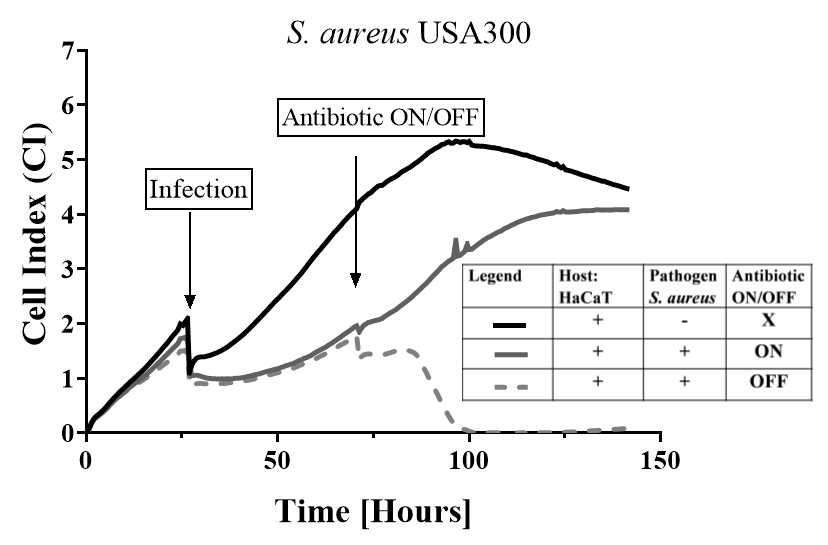

Supplement: Supplementary file 3 — Supplementary Material 3 [file 41598_2024_84312_MOESM3_ESM.tif]

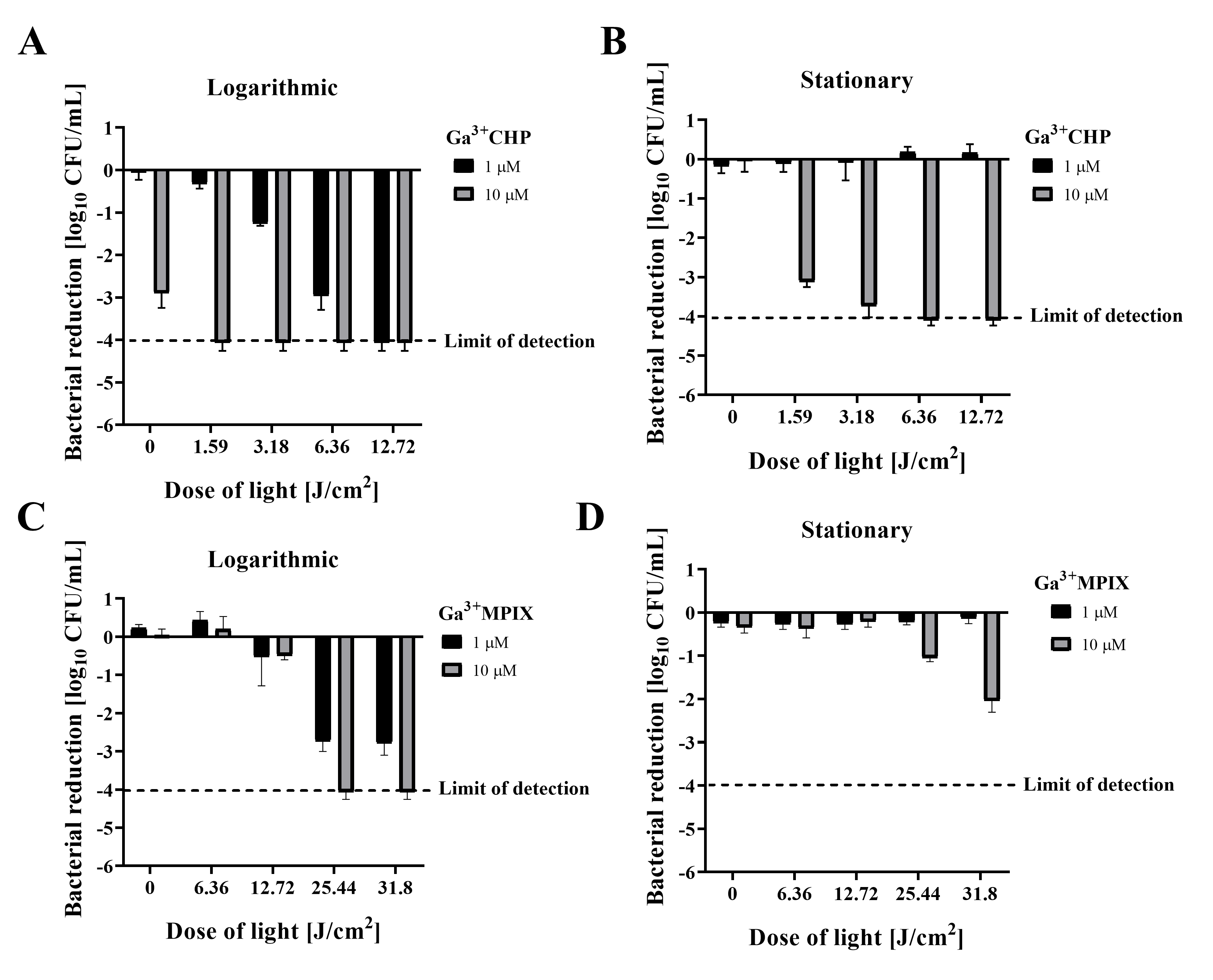

Supplement: Supplementary file 4 — Supplementary Material 4 [file 41598_2024_84312_MOESM4_ESM.tif]

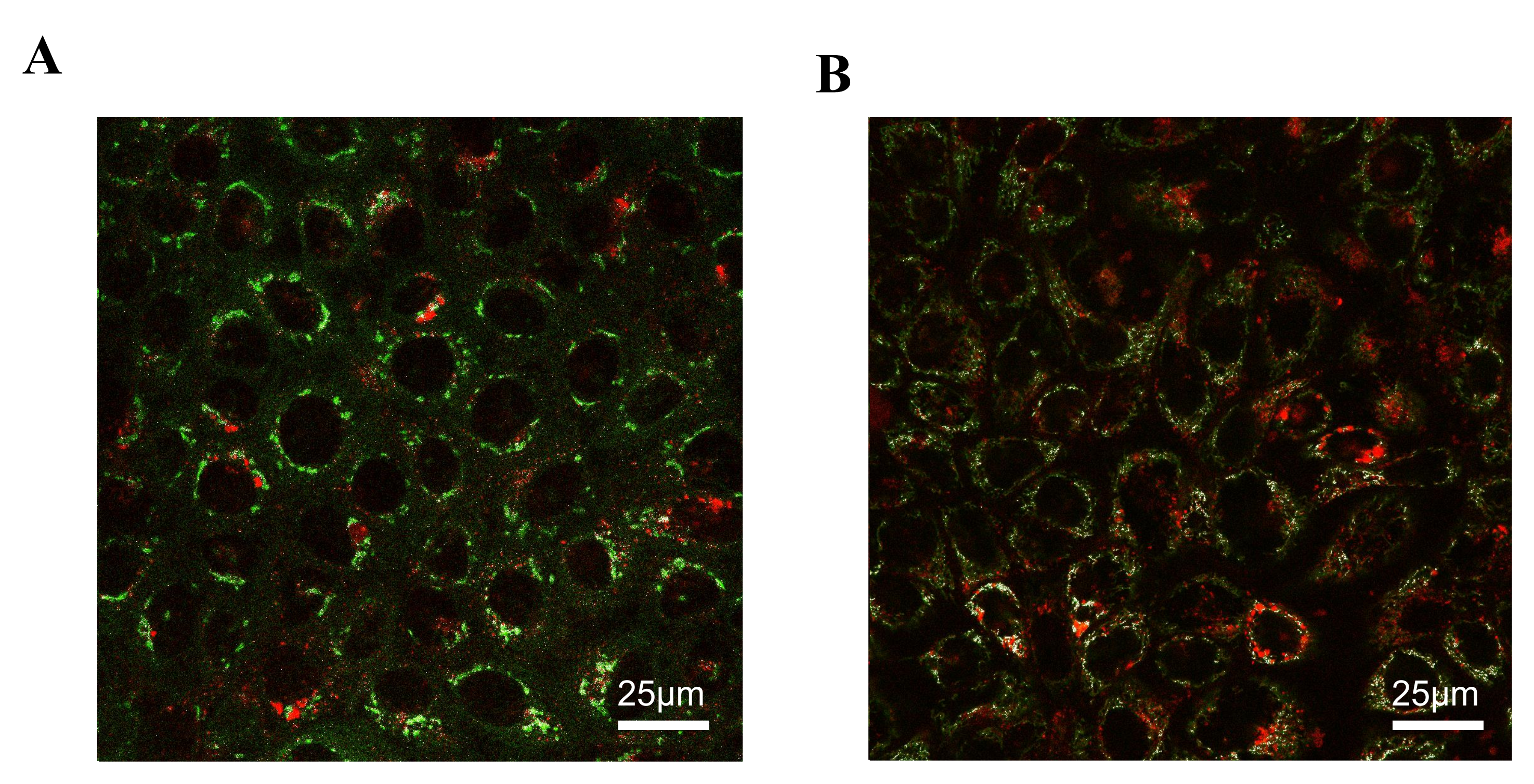

Supplement: Supplementary file 5 — Supplementary Material 5 [file 41598_2024_84312_MOESM5_ESM.tif]

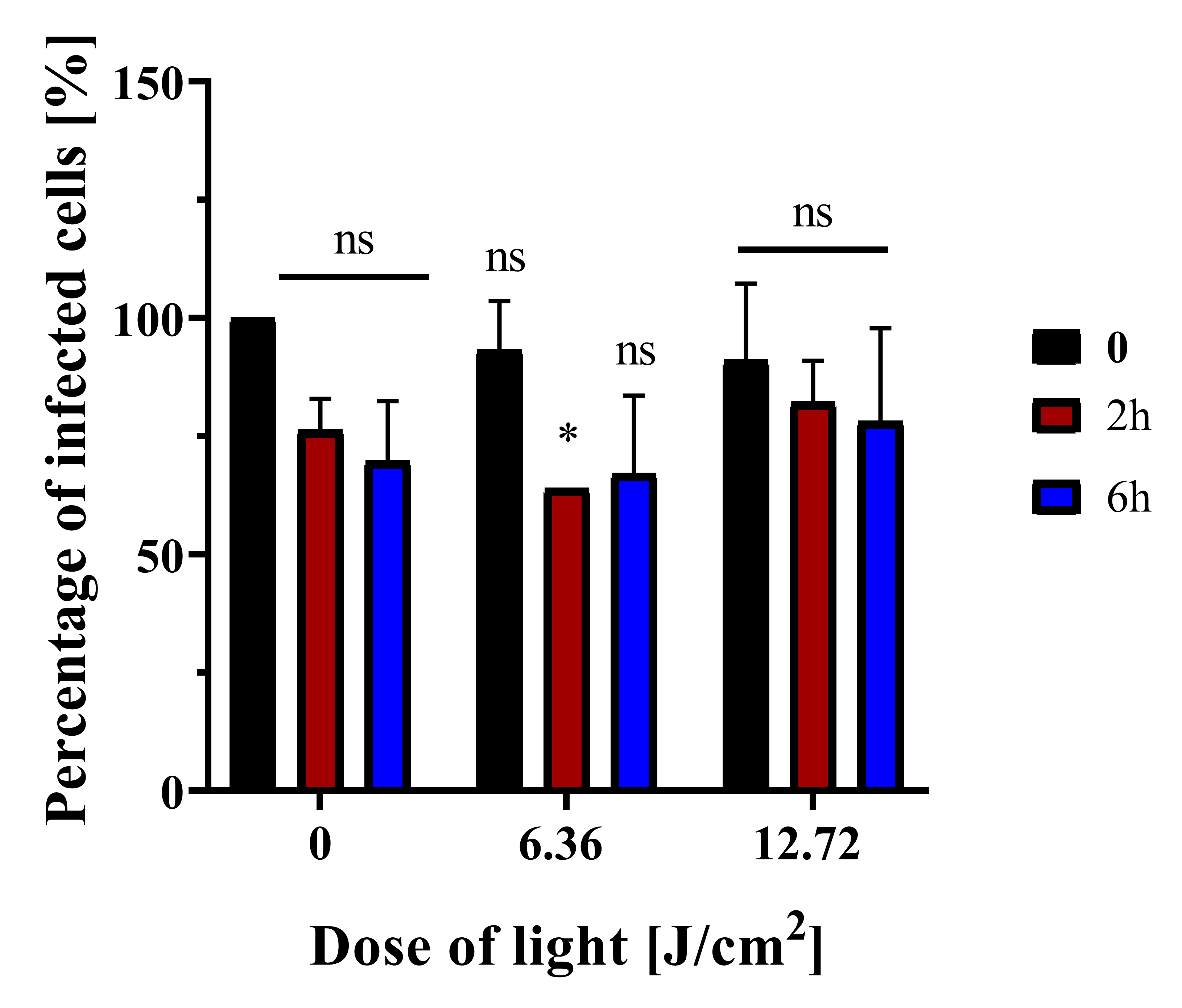

Supplement: Supplementary file 6 — Supplementary Material 6 [file 41598_2024_84312_MOESM6_ESM.tif]
