# Supplementary material for: Harnessing light-activated gallium porphyrins to combat intracellular Staphylococcus aureus using an in vitro keratinocyte infection model
Source: Sci Rep. 2025 Jan 8;15:1295. doi: 10.1038/s41598-024-84312-4 (PMC11711192; doi:10.1038/s41598-024-84312-4)
Supplement: Supplementary file 7 — Supplementary Material 7 [file 41598_2024_84312_MOESM7_ESM.docx]

**Supporting information captions**

**Harnessing light-activated gallium porphyrins to combat intracellular *Staphylococcus aureus* using an *in vitro* keratinocyte infection model.**

Klaudia Szymczak^1^, Michał Rychłowski^2^, Lei Zhang^3^, Joanna Nakonieczna^1*^

^1^ Laboratory of Photobiology and Molecular Diagnostics, Intercollegiate Faculty of Biotechnology, University of Gdansk and Medical University of Gdansk, Poland,

^2^ Laboratory of Virus Molecular Biology, Intercollegiate Faculty of Biotechnology, University of Gdansk and Medical University of Gdansk, Poland,

^3^ Department of Biochemical Engineering, School of Chemical Engineering and Technology, Frontier

Science Center for Synthetic Biology and Key Laboratory of Systems Bioengineering (MOE), Tianjin University, China

We developed a model of keratinocyte infection and characterized the process of *S. aureus* internalization into keratinocytes. To confirm the presence of intracellular *S. aureus* after infection, fluorescence microscopy images were taken on the first day after infection. The green fluorescent protein (GFP) signal from the *S. aureus* USA300 bacteria (white arrow) was observed inside the host cell along with the nuclei, which were stained with HOECHST dye (blue signal, black arrow) (**Fig S1 A**). To confirm the intracellular presence of the pathogen, three-dimensional images were taken using scanning fluorescence microscopy (**Fig S1 B**), confirming that bacteria were intracellularly localized. Then, we examined the effect of various multiplicities of infection (MOI) (0-100) on the viability of *S. aureus* in medium and inside keratinocytes by studying three fractions after infection. The fractions were as follows: (i) extracellular, (ii) intracellular, and (iii) intracellular + adherent *S. aureus* (**Fig S1 C**). The number of extracellular *S. aureus* collected from the culture medium increased with the higher MOI used for infection, although the data were not statistically significant. In the case of intracellular *S. aureus*, the viability was estimated at 5.6 log_10_ CFU/mL for MOI 100, which was much higher than 4.8 log_10_ for MOI 10, and 4.7 log_10_ for MOI 1 (**Fig S1 D**). By measuring the GFP signal derived from the *S. aureus* strain, a higher number of infected cells was observed for both MOIs 10 and 100 than for an MOI of 1. However, there was no significant difference in the rate of intracellular invasion between the MOI 10 and 100 inoculum according to the flow cytometry studies (**Fig S1 E**). The *S. aureus* infection delayed the growth rate of HaCaT cells. However, host cells harboring intracellular *S. aureus* can grow and proliferate, however there is no significant difference in the rate of intracellular invasion between MOI10/100 (Fig S1D/E), and host cell proliferation curves are similar for MOI1/10 (Fig S1F).

**
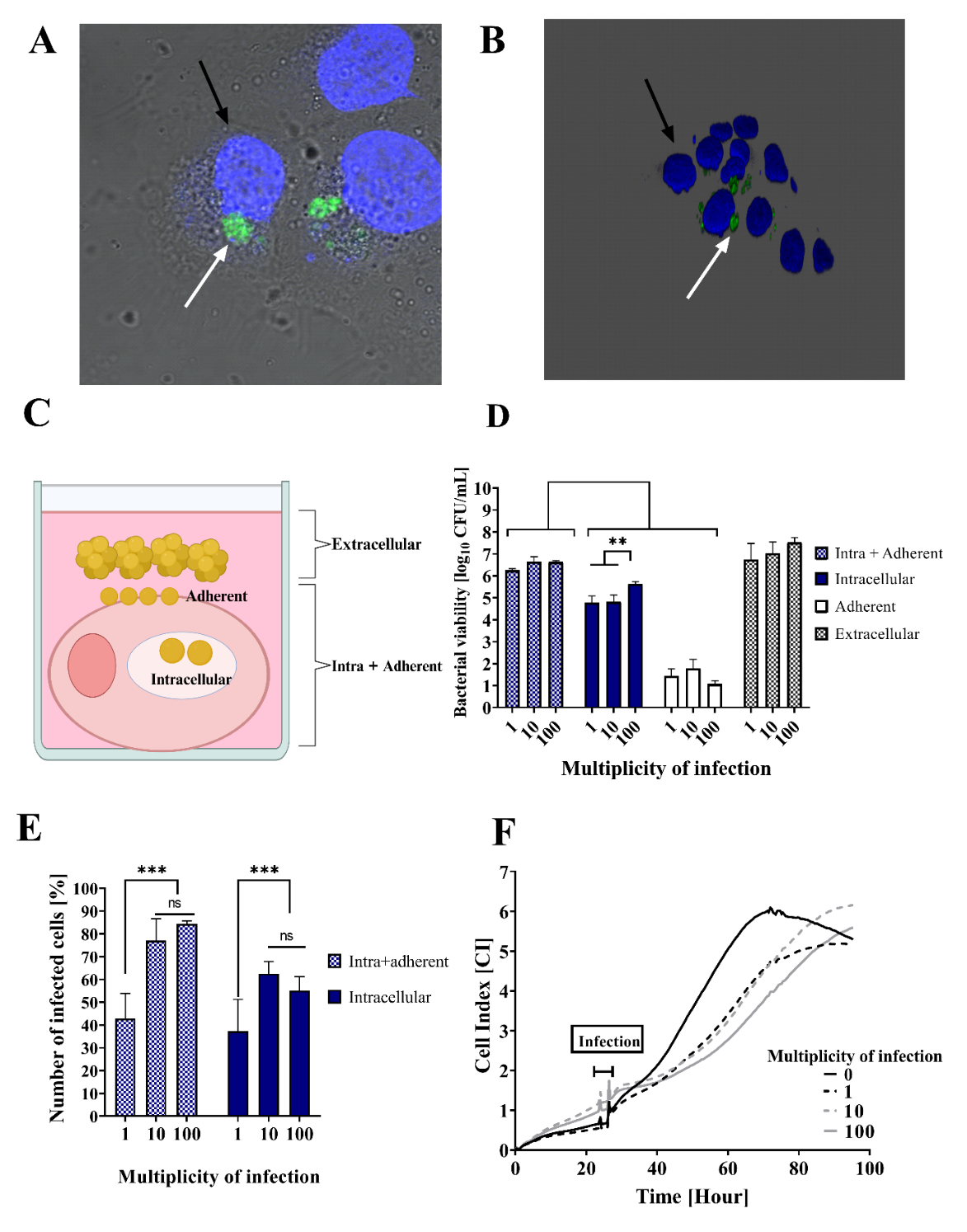
**

**Fig S1**. **Effect of *S. aureus* multiplicity of infection (MOI) on internalization, infection, and keratinocyte growth.**

**A)** Keratinocytes **(**HaCaT cell line) infected with *S. aureus* USA300 (green signal, white arrow). The host cell nucleus was stained (blue signal, black arrow). **(B)** 3D image of the coculture of HaCaT and *S. aureus* USA300. **(C)** Schematic representation of bacterial fractions collected after each step of coculture preparation. ‘Extracellular’ refers to free-floating *S. aureus* collected from the growth medium after a 2-hour staphylococcal infection; ‘Adherent’ - *S. aureus* attached to host cell; ‘Intracellular’ - *S. aureus* accumulated inside host cell; ‘Intra+Adherent’ – the combined number of *S. aureus* in adherent and intracellular fraction. (**D)** Bacterial viability was determined by seeding bacteria onto agar plates and counting colony-forming units (CFU/mL) in each collected fraction. Different ratios of the number of bacteria to the number of host cells were used (MOI 1-100) for infection model preparation. The significant differences are marked with asterisks [**p < 0.01] (two-way Anova) (**E)** Percentage of infected, GFP-expressing cells collected immediately after infection (MOI 0-100) (‘Intra+Adherent’ fraction) or after 1-hour antibiotic exposure (‘Intracellular’ fraction). The GFP signal was measured by flow cytometry. Significant differences in infected cell viability between tested samples at the respective p values are marked with asterisks [***p < 0.001] and calculated with respect to uninfected cells at each time point (two-way Anova). (**F**) Real-time host growth analysis after infection with *S. aureus* USA300 at MOI 0-100. After 2 hours, the medium was removed, the cells were washed, and antibiotics were added to ensure the intracellular maintenance of *S. aureus*.

**Movie S1. Cell death of infected HaCaT cell under antibiotic pressure (Antibiotic ON)**Fluorescent *S. aureus* USA300 bacteria (green signal), HaCaT cells Transmitted Light (gray signal). 17-hour time lapse microscopy analyze, 15-min interval.

**The behavior of intracellular *S. aureus* is strain-dependent.**

To examine whether the staphylococcal invasion into keratinocytes is dependent on the *S. aureus* strain, we compared keratinocyte infections with two *S. aureus* strains: the hypervirulent USA300 strain and the nonvirulent strain RN4220. First, we examined the intracellular viability of the two bacterial strains during infection and their persistence under antibiotic pressure for several days after infection. The nonvirulent RN4220 strain exhibited higher intracellular viability, as shown in a several-day culture model, than the hypervirulent USA300 (5.8 log_10_ vs. 4.2 log_10_ CFU/mL on the 1^st^ day postinfection) (**Fig S2 A**). RN4220 remained within the keratinocytes longer than USA300, which was completely titrated out of the cells (reaching the detection limit of 2 log_10_) on day 5 postinfection. The difference in infection between the two strains was also evident in the host growth rate (**Fig S2 B**). RN4220 reduced host cell growth after infection significantly more than USA300. Nevertheless, host cells intracellularly harboring bacteria could grow and proliferate (**Fig S2C and D**). Interestingly, when antibiotic pressure was removed and the medium was changed to a nonantibiotic medium (Antibiotic OFF) after the 1^st^ day postinfection (dashed gray line), *S. aureus* RN4220 continued to persist intracellularly and did not leave the host cells to cause recurrent infection, while USA300 was released from the host, causing cell death, restoring extracellular infection, and inducing significant toxicity. A similar effect was observed when the antibiotic pressure was maintained longer (up to the 3^rd^ day postinfection) for USA300 (**Fig S3**), showing that even the low-viability intracellular inoculum was able to escape and resume the infection. We observed the behavior of *S. aureus* in the infection process, including intracellular persistence and the ability to escape from the host for reinfection, is strain-dependent.


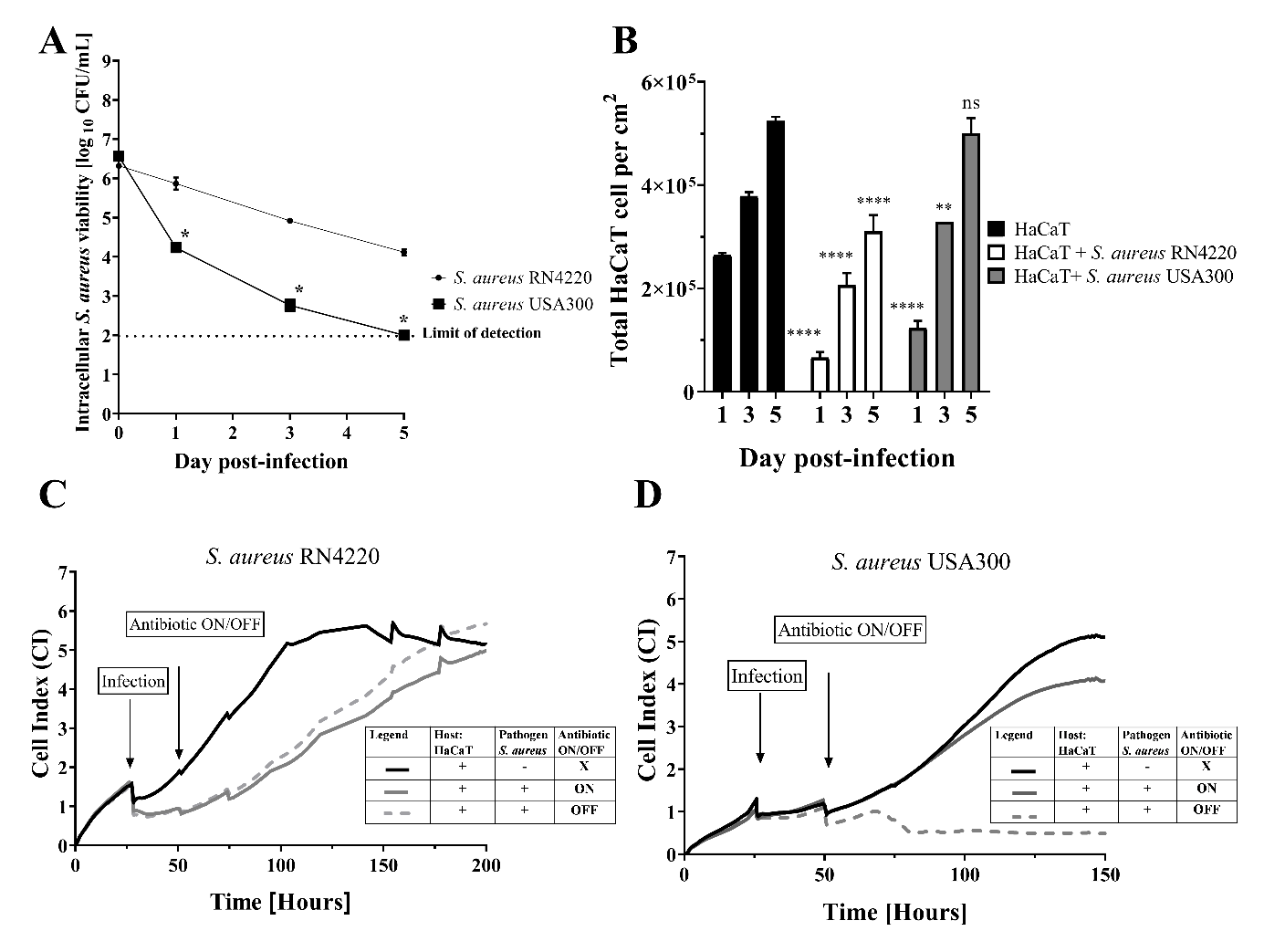


**Fig S2**. **Characterization of intracellular invasion of hypervirulent (USA300) and nonvirulent (RN4220) *S. aureus* strains in an infection model over time.**

**(A)** Intracellular viability of the two *S. aureus* strains over time after infection (1-5 days). Significant differences in USA300 viability at the tested time points were calculated relative to the reference strain RN4220 (BEI Resources, USA) with corresponding p values marked with asterisks [*p < 0.05] **(B)** HaCaT cell viability over time (1-5 days) after infection with two tested *S. aureus* strains. The significant differences in infected cell viability at the respective p values are marked with asterisks [*p < 0.05; **p < 0.01; ***p < 0.001] and were normalized using the uninfected cells at each studied time point (two-way Anova). Infection of HaCaT cells (**A, B**) in medium without antibiotics was performed at an MOI of 10 for 2 hours; then, the cells were cultured under antibiotic pressure until the end of the experiment. **(C, D)** Real-time analysis of HaCaT cell growth after infection with *S. aureus* RN4220 (**C**) or USA300 (**D**). HaCaT cell infection was performed at an MOI of 10 for 2 hours; no antibiotic (X), culture under antibiotic pressure (Antibiotic ON) or antibiotic removal (Antibiotic OFF), which was determined on day 1 postinfection. Bacterial strains were grown at 37 °C with 10 μg/mL of chloramphenicol for USA300 or trimethoprim for RN4220 to sustain the GFP plasmid.

**
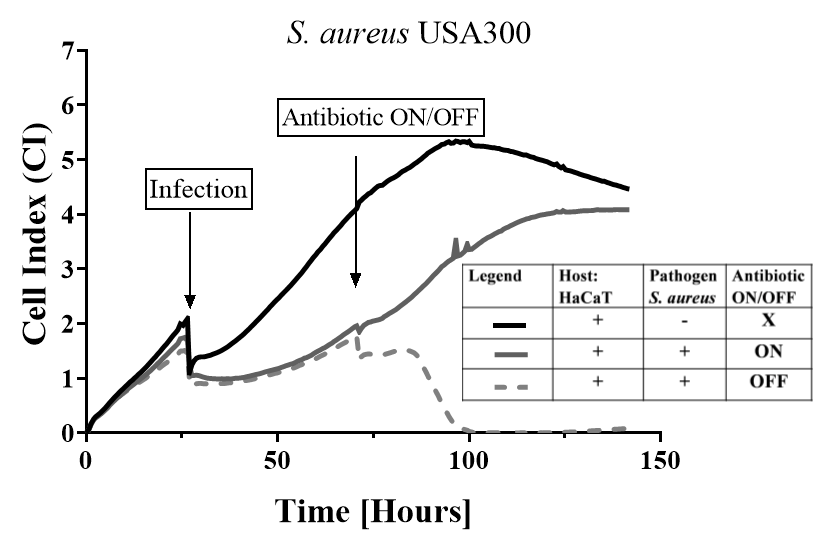
**

**Fig S3. Recurrent infection of *S. aureus* USA300 at 3^rd^ day post-infection**

Infection of HaCaT cells in a medium without antibiotics was carried out at an MOI of 10 for 2 hours, then the cells were cultured under antibiotic pressure (Antibiotic ON) until the medium was changed to with or without antibiotic (Antibiotic OFF) at 3^rd^ day post-infection.

**
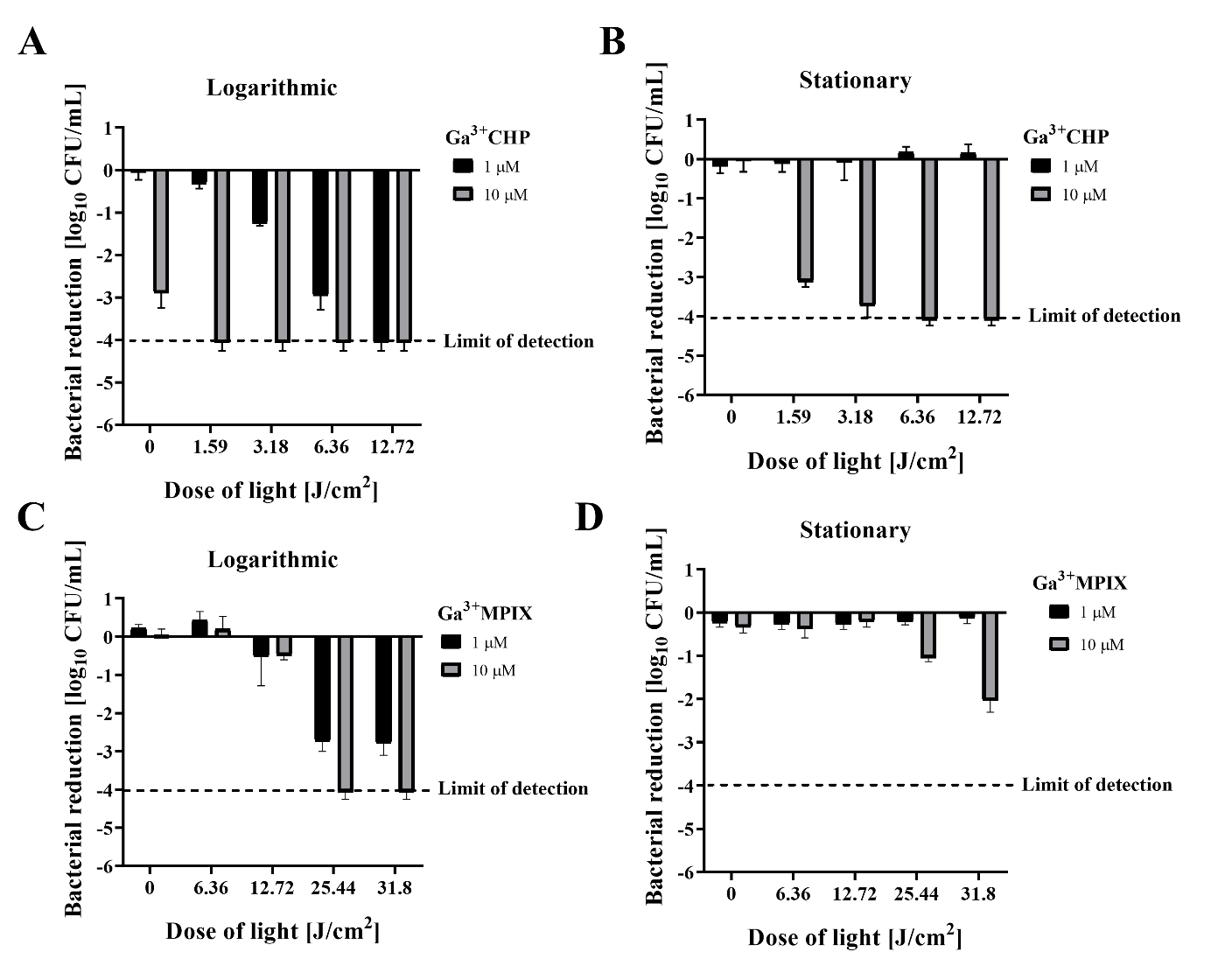
**

**Fig S4. Photoinactivation of *S. aureus* USA300 with gallium metalloporphyrins at bacterial logarithmic or stationary growth phase.**

Two photosensitizers, Ga^3+^CHP (**A, B**) and Ga^3+^MPIX (**C, D**), activated with green (522 nm) light were used to evaluate the *S. aureus* USA300 susceptibility to aPDI during logarithmic (**A, C**) or stationary phase (**B, D**) of growth. The bacterial reduction was calculated with respect to the untreated cells. Each experiment was performed in three independent biological replicates. The data are presented as the mean ± SD of three separate experiments.

**
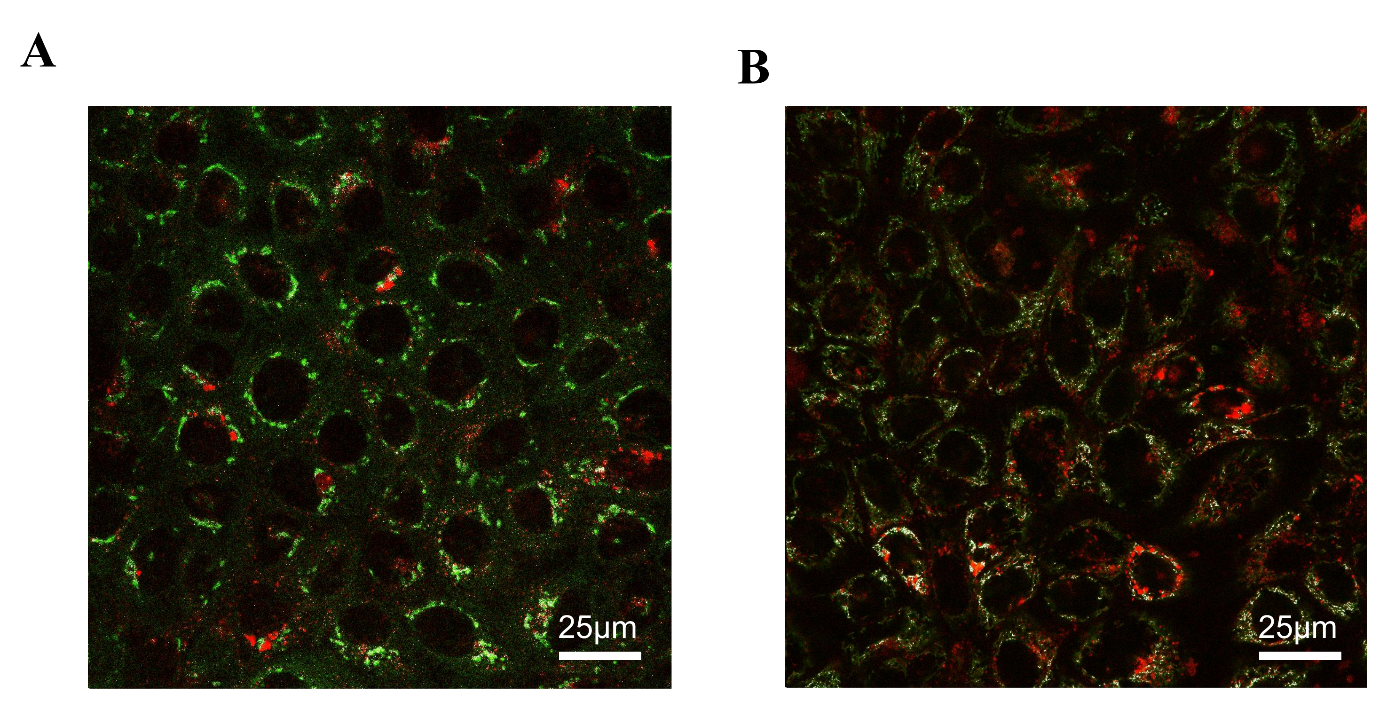
**

**Fig S5. Colocalization of intracellular Ga^3+^CHP in Golgi apparatus and mitochondria.**

Intracellular localization of Ga^3+^CHP in human keratinocytes after 6 hours of incubation. Analysis was performed with compounds’ colocalization in the Golgi apparatus (A) and mitochondria (B). The red signal represents Ga^3+^CHP, while the green signal is from either Golgi (A) or mitochondria (B). Areas of colocalization are marked in white. All colocalization parameters are detailed in the **Table S1**.

**Table S1 Coefficients of Ga^3+^CHP accumulation in the specific cellular compartments**

| **Type accumulation** | **The Pearson’s coefficient** | **Overlap coefficient** |
| --- | --- | --- |
| **Ga^3+^CHP + Lysosome** | 0.4695 | 0.65 |
| **Ga^3+^CHP + Golgi apparatus** | 0.18 | 0.44 |
| **Ga^3+^CHP + Mitochondria** | 0.39 | 0.61 |

The results are reported as Pearson correlation coefficients and colocalization rates (%). The Pearson’s coefficient ranges from -1 to +1 with a statistically significant score colocalization value above 0.5. The overlap factor ranges from 0 to 1 with co-localization values ​​above 0.6. The quantitative analysis of colocalization was performed with Leica Application Suite X version 3.5.2.18963.

| **Type of image** | | **The Pearson’s coefficient** | **Overlap coefficient** |
| --- | --- | --- | --- |
| **Ga^3+^CHP + Lysosome** | | 0.4695 | 0.65 |
| ***S. aureus* + Lysosome** | | 0.19 | 0.3064 |
| ***S. aureus* + Ga^3+^CHP** | | 0.5085 | 0.6 |
| ***S. aureus* +Ga^3+^CHP + Lysosome** | ***S. aureus* +Ga^3+^CHP** | 0.67 | 0.7 |
|  | ***S. aureus +* Lysosome** | 0.6145 | 0.6352 |
|  | **Ga^3+^CHP + Lysosome** | 0.6759 | 0.7563 |

**Table S2** **Coefficients of Ga^3+^CHP and *S. aureus* colocalization within lysosomal structures of human keratinocytes**

The results are reported as Pearson correlation coefficients and colocalization rates (%). The Pearson’s coefficient ranges from -1 to +1 with a statistically significant score colocalization value above 0.5. The overlap factor ranges from 0 to 1 with co-localization values ​​above 0.6. The quantitative analysis of colocalization was performed with Leica Application Suite X version 3.5.2.18963


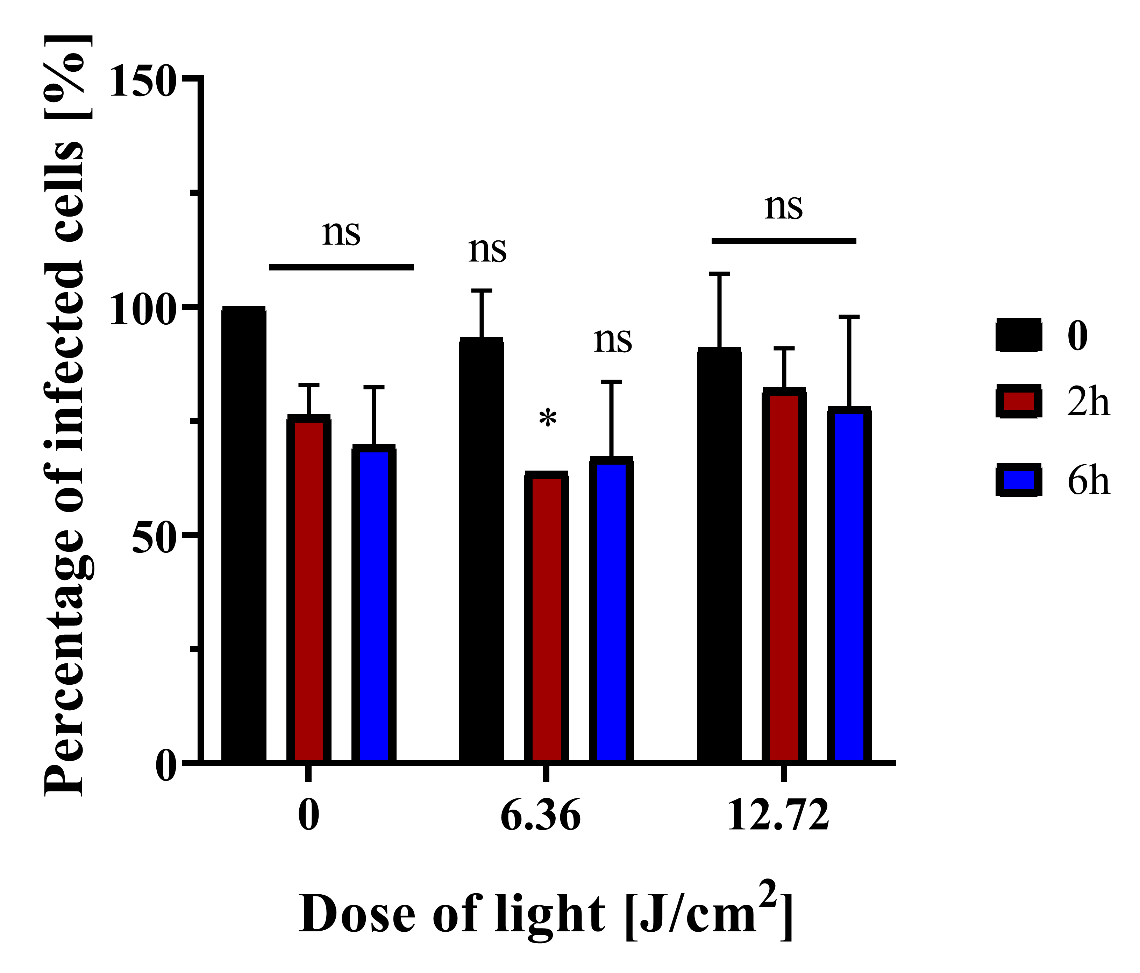


**Figure S6 Light-activated Ga^3+^MPIX has no impact on the number of GFP-expressed cells.**

Percentage of infected cells after aPDI with Ga^3+^MPIX. The number of GFP-expressing cells after 2- or 6-hour dark incubation followed with either dark treatment or green light illumination. Cells were collected, fixed and GFP signal was measured by flow cytometry. All results were calculated in reference to the untreated control (cells with no compound and no light exposure) and analyzed using two-way Anova.
